# The interplay of autophagy and oxidative stress in the pathogenesis and therapy of retinal degenerative diseases

**DOI:** 10.1186/s13578-021-00736-9

**Published:** 2022-01-03

**Authors:** Kun-Che Chang, Pei-Feng Liu, Chia-Hsuan Chang, Ying-Cheng Lin, Yen-Ju Chen, Chih-Wen Shu

**Affiliations:** 1 Department of Ophthalmology and Neurobiology, Louis J. Fox Center for Vision Restoration, University of Pittsburgh School of Medicine, Pittsburgh, PA USA; 2 Graduate Institute of Medicine, College of Medicine, Kaohsiung Medical University, Kaohsiung, Taiwan; 3 Department of Biomedical Science and Environmental Biology, PhD Program in Life Science, College of Life Science, Kaohsiung Medical University, Kaohsiung, Taiwan; 4 Department of Medical Research, Kaohsiung Medical University Hospital, Kaohsiung, Taiwan; 5 Center for Cancer Research, Kaohsiung Medical University, Kaohsiung, Taiwan; 6 Institute of BioPharmaceutical Sciences, National Sun Yat-Sen University, No. 70, Lianhai Rd., Gushan Dist., Kaohsiung, 80424 Taiwan; 7 Division of Gastroenterology and Hepatology, Department of Internal Medicine, Taichung Veterans General Hospital, Taichung, Taiwan; 8 Institute of Clinical Medicine, National Yang Ming Chiao Tung University, Taipei, Taiwan; 9 Department of Medical Research, Taichung Veterans General Hospital, Taichung, Taiwan; 10 Division of Allergy, Immunology and Rheumatology, Department of Internal Medicine, Taichung Veterans General Hospital, Taichung, Taiwan

**Keywords:** Autophagy, Reactive oxygen species, Glaucoma, Age-related macular degeneration, Diabetic retinopathy, Optic nerve atrophy

## Abstract

Oxidative stress is mainly caused by intracellular reactive oxygen species (ROS) production, which is highly associated with normal physiological homeostasis and the pathogenesis of diseases, particularly ocular diseases. Autophagy is a self-clearance pathway that removes oxidized cellular components and regulates cellular ROS levels. ROS can modulate autophagy activity through transcriptional and posttranslational mechanisms. Autophagy further triggers transcription factor activation and degrades impaired organelles and proteins to eliminate excessive ROS in cells. Thus, autophagy may play an antioxidant role in protecting ocular cells from oxidative stress. Nevertheless, excessive autophagy may cause autophagic cell death. In this review, we summarize the mechanisms of interaction between ROS and autophagy and their roles in the pathogenesis of several ocular diseases, including glaucoma, age-related macular degeneration (AMD), diabetic retinopathy (DR), and optic nerve atrophy, which are major causes of blindness. The autophagy modulators used to treat ocular diseases are further discussed. The findings of the studies reviewed here might shed light on the development and use of autophagy modulators for the future treatment of ocular diseases.

## Background

Christian de Duve, a Nobel Prize winner in 1974, observed cellular autophagic structures by electron microscopy sixty years ago due to the discovery of peroxisomes and lysosomes [1, 2]. In the early 1990s, the Japanese scientist Yoshinori Ohsumi identified the autophagy-related (ATG) genes required for autophagosome formation and explained how eukaryotic cells recycle their components [3–6]. Autophagy can recruit damaged proteins/organelles to lysosomes through selective adaptors or non-selective bulk degradation to generate different substrates, such as nucleotides, sugars, fatty acids, and amino acids, for new synthesis [6, 7]. Oshumi’s findings opened up research on the role of autophagy in the physiology of normal cells and the pathogenesis of various diseases and conditions, including neurodegenerative diseases, infections, and cancer. Therefore, Yoshinori Ohsumi was awarded the Nobel Prize in Physiology or Medicine in 2016.

There are three major types of autophagy: macroautophagy, microautophagy, and chaperone-mediated autophagy (CMA). Although crosstalk may occur among the three pathways, and all three pathways deliver components to lysosomes for degradation, the mechanisms of delivery are quite different among them.

### Macroautophagy

Since macroautophagy is the most common form of autophagy, autophagy usually means macroautophagy. Macroautophagy requires autophagosome formation to pack abnormal proteins and organelles into autophagosomes and fusion with lysosomes to digest the contents [8]. Most forms of macroautophagy are selective because specific cargos or adaptors are essential for the recruitment of targets to autophagosomes, such as in mitophagy and pexophagy for the degradation of mitochondria and peroxisomes, respectively [8–11]. Several adapters for selective autophagy, such as squestome (SQSTM1, also known as p62) and NBR1, associate with ubiquitinated cargo proteins and autophagosomal protein LC3 via ubiquitin-associated (UBA) and LC3-interacting region (LIR) motif, respectively [12].

### CMA

CMA is a chaperone (HSC70)-dependent degradation pathway. HSC70 recognizes cytosolic unfolded proteins containing KFERQ pentapeptide and delivers it to lysosomes by binding with the transmembrane receptor LAMP-2A [13, 14]. HSP90 then associates with the LAMP-2A complex to assist translocated substrate proteins into lysosomes for degradation [15]. The LAMP-2A complex is further disassembled to a monomer and eventually degraded by cathepsin A and a metalloproteinase in the lipid microdomain [16].

### Microautophagy

Microautophagy was defined as micro portion of lysosomal membrane to engulf autophagic cargos, including proteins and organelles, in cells [17]. Microautophagy can be classified into three types according to the morphology of membrane deformation: type 1, lysosomal protrusion; type 2, lysosomal invagination; and type 3, endosomal invagination [18]. Some ATG proteins or HSC70 or ESCRT proteins are involved in the membrane deformation process [19, 20]. Therefore, crosstalk may occur among microautophagy and macroautophagy, CMA and endocytosis. Thus, further studies are required to elucidate the potential mechanisms through which this crosstalk would occur.

## Autophagy-related proteins

**Fig. 1:**
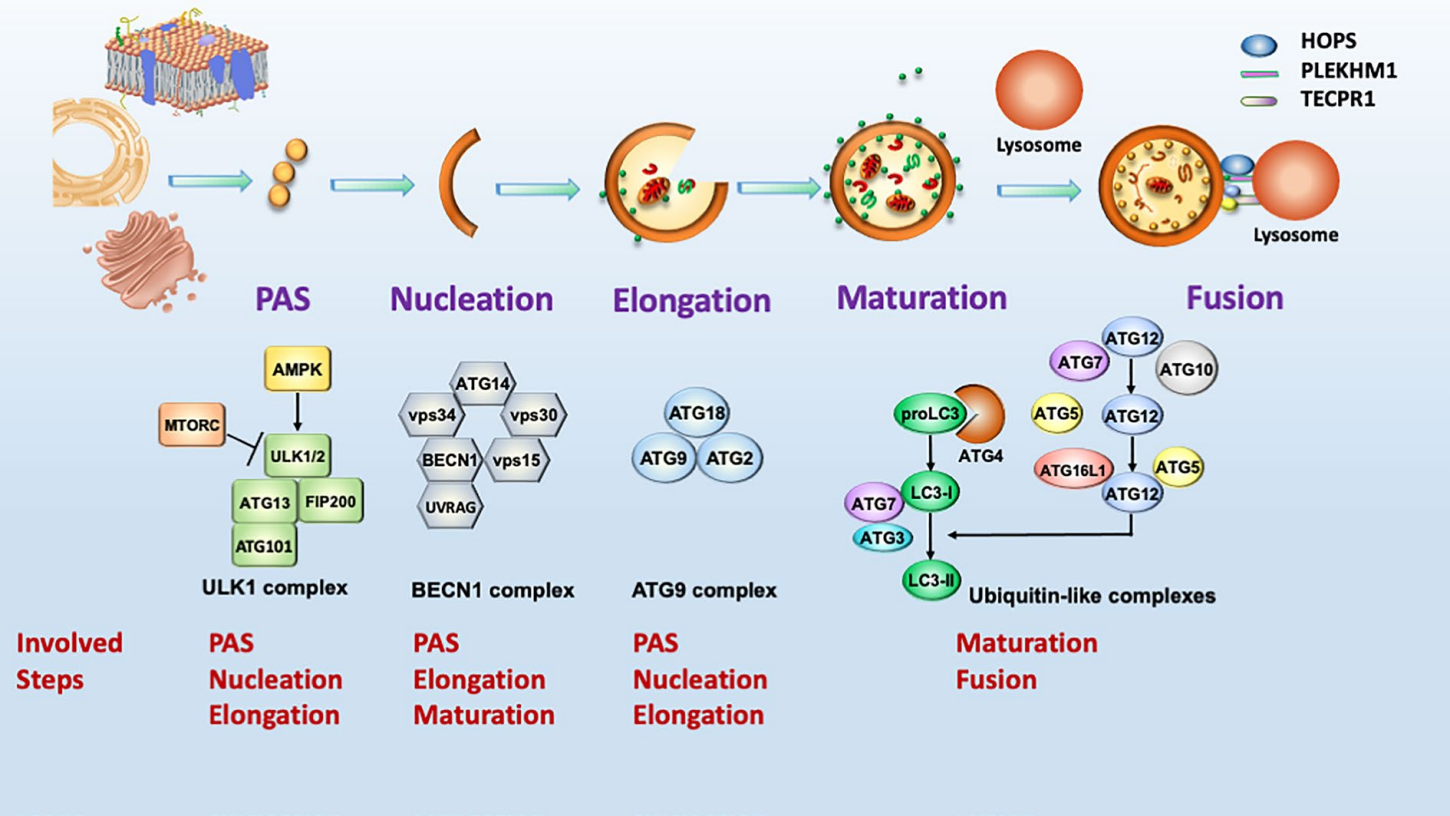
Schematic diagram for components of each ATG-mediated complex and their involvement in autophagy steps. ULK1/2, BECN1, ATG9 and ubiquitin-like (LC3 and ATG12)-mediated complexes are the four major complexes involved in the core machinery of autophagy, from the PAS to autophagosome maturation/fusion. Many complexes are involved in several stages of autophagy, such as the ULK1/2-mediated complex involved in the PAS, autophagosome nucleation and elongation, since ULK1/2 can phosphorylate and activate many components of the other complexes. AMPK and MTORC are positive and negative regulators of ULK1/2, respectively. Moreover, LC3 and ATG12 are also involved in tethering complexes for the specificity of autophagosomal fusion with lysosomes

There are more than 40 Atg proteins involved in macroautophagy signaling in yeast cells. Most of the proteins have been found to have ATG homologous proteins in mammalian cells, in which about 20 ATG proteins play crucial roles in autophagy progression, including pre-autophagosomal structure (PAS) formation, autophagosome maturation and fusion with lysosomes. The ATG proteins can be clustered into four complexes as listed in Table 1 [21–25] and their functions are described as below. The involvement of these complexes in autophagy machinery is also shown in Fig. 1.

i) ULK1/2-containing complex- ULK1/2 is the only ATG kinase that binds and phosphorylates FIP200 (mammalian Atg17 homologous), ATG13 and ATG101 for autophagosome nucleation and formation [26, 27]. ULK1/2 also phosphorylates several ATG proteins, such as ATG9 at Ser14, ATG4B at Ser316, BECN1 at Ser14 and ATG14L at Ser29 [28]. Moreover, AMPK directly phosphorylates ULK1, particularly in S317 and S777, to activate its kinase activity, whereas MTORC1 directly phosphorylates ULK1 at S757 to block the binding between AMPK and ULK1 [29]. Interestingly, ULK1 can phosphorylate and negatively regulate both AMPK and MTORC1 activity, suggesting the regulation loop of AMPK-MTORC1-ULK1 are important to control autophagic activity for maintaining energy homeostasis.
ii) BECN1-containing complex- BECN1 attaches to VPS15 and VPS34, which is a lipid kinase class III phosphatidylinositol 3 kinase (PI3K) that triggers the phosphorylation of phosphatidylinositol and results in phosphatidylinositol 3-phosphate (PI3P) formation [30, 31]. ATG14L/Barkor (mammalian Atg14 homologous) recruits the complex to the PAS site. UV radiation resistance-associated (UVRAG) protein associates with the BECN1 complex for autophagosome formation and maturation [32, 33]. In addition, AMPK phosphorylates BECN1 to activate VPS34 activity and induce autophagy [34], whereas Run domain Beclin-1 interacting and cysteine-rich containing (Rubicon) binds to BECN1 and inactivate class III PI3K complex 2 for blocking fusion step between autophagosome and lysosome [35].
iii) ATG9-containing complex- ATG9 is only one transmembrane protein among ATG proteins. ATG9A forms a homotrimer to form a pore to translocate ATG2-delivered phospholipids for PAS formation and phagophore nucleation [36]. ATG9 also coordinates with the ATG9 receptor and ATG11 to recruit ATG2, WIPI1/2 (mammalian Atg18 homologous) and LC3 for lipid transfer, which is important for autophagosome expansion [37, 38].
iv) ATG12 and LC3 ubiquitin-like conjugation complexes- ATG12 conjugated to ATG5 and LC3/GABARAP conjugated to phosphatidylethanolamine (PE) (LC3-II/GABARAP-II) are two ubiquitin-like complexes are essential for autophagosome elongation and maturation in mammalian cells [39, 40]. LC3 and GABARAP are activated by ATG4 family proteases (including ATG4A, ATG4B, ATG4C and ATG4D) before conjugation [41]. All the conjugation requires the E1-like enzyme ATG7 and the E2-like enzyme ATG10 (for ATG12-ATG5) or ATG3 (for LC3-II/GABARAP-II) [42]. ATG16L stabilizes ATG12-ATG5 conjugates to form a complex of approximately 800 kDa and serves as an E3-like enzyme for the conjugation.

**Table 1:** The functions of each component in the autophagy complex involved in the autophagy machinery

Complex	Components		Functions
	Yeast	Mammals	
Atg1/ULK1/2 complex	Atg1	ULK1/2	It is the only ATG protein with kinase activity and phosphorylates several other ATG proteins (ATG9, BECN1, ATG14L) for the PAS, autophagosome elongation and maturation
	Atg13	ATG13	It serves as a linker among ULK1/2, FIP200 and ATG101
	Atg17	RB1CC1/FIP200	It is a scaffold protein for ULK1/2 and ATG13 and serves as a scaffold protein for the ULK1/2 complex
	–	ATG101	It interacts with ATG13
BECN1 complex	Atg6	Beclin1	It is a core component in class III PI3KI/II and binds lipids. It also associates with UVRAG for autophagosome elongation and maturation
		VPS34	It is a catalytic subunit of class III PI3K to generate PI3P
		VPS15	It is a protein kinase involved in the PI3P pathway
	Atg14	ATG14L (Barkor)	It associates with the BECN1 complex for membrane targeting
ATG9A complex		ATG9A	It is the only transmembrane protein among ATG proteins and forms homotrimer for the PAS, nucleation and autophagosome formation
	Atg18	WIPI1/2	It attaches to PI3P for the transportation of ATG9
	Atg2	ATG2A	It attaches to WIPW1/2
Ubiquitin-like complex	Atg8	LC3A-C, GABARAP	It is a ubiquitin-like protein and ligates with PE for autophagosome elongation and sealing
	Atg12	ATG12	It is another ubiquitin-like protein and ligates with ATG5 to form an E3-like ligase with ATG16
	Atg4	ATG4A-D	It is a protease required for the cleavage and activation of proLC3/GABARAP at the C-terminus for conjugation and further deconjugation of LC3/GABARAP-PE
	Atg7	ATG7	It serves as an E1-like enzyme for LC3 and ATG12 conjugation
	Atg3	ATG3	It serves as an E2-like enzyme for LC3/GABARAP conjugation with PE
	Atg10	ATG10	It serves as an E2-like enzyme for ATG12 conjugation with Atg5
	Atg5	ATG5	It covalently binds to ATG12 and associates with ATG16 to form the E3-like enzyme complex
	Atg16	ATG16L1	It is a part of the E3-like enzyme complex along with ATG12 and ATG5

In addition to autophagosome maturation, LC3 and ATG12 ubiquitin-like proteins are also involved in the tethering complex. The tethering complex for the fusion step between autophagosomes and lysosomes consists of the homotypic fusion and protein sorting (HOPS) complex, Rab7, adaptors and receptors (LC3-II/ATG12-ATG5) [43]. The HOPS complex consists of Vps16, Vps18, Vps33, Vps39, and Vps41 and connects to syntaxin 17 by binding with oligomeric ATG14L to mediate fusion [44, 45]. PLEKHM1 and TECPR1 are adaptor proteins that connect autophagosomal LC3-II and ATG12-ATG5 with lysosomal Rab7 to ensure fusion specificity [43].

## Reactive oxygen species (ROS) and autophagy

Oxidative stress is highly associated with elevated intracellular reactive oxygen species (ROS), which are involved in cellular physiological regulation and the pathogenesis of diseases, such as neuronal, ocular and cardiovascular diseases [46–48]. Intracellular ROS are mainly (approximately 90%) generated by the electron transport chain in the inner membrane of mitochondria and consist of H_2_O_2_, superoxide (O_2·_−) and hydroxyl radicals (OH_·_) [49, 50]. ROS can oxidize organelles, nucleic acids, proteins and lipids, which results in cellular damage [51]. ROS not only trigger the autophagy pathway to maintain redox homeostasis and remove oxidized organelles and other components [52] but also inhibit autophagy, likely directly oxidizes ATG proteins (ATG7 and ATG10) or inactivating autophagy modulators (TFEB and PTEN) [53–55]. Conversely, autophagy can modulate ROS levels through several mechanisms. The reciprocal regulation of ROS and autophagy are discussed below.

### The regulation of autophagy by ROS

#### ROS induce autophagy

**Fig. 2:**
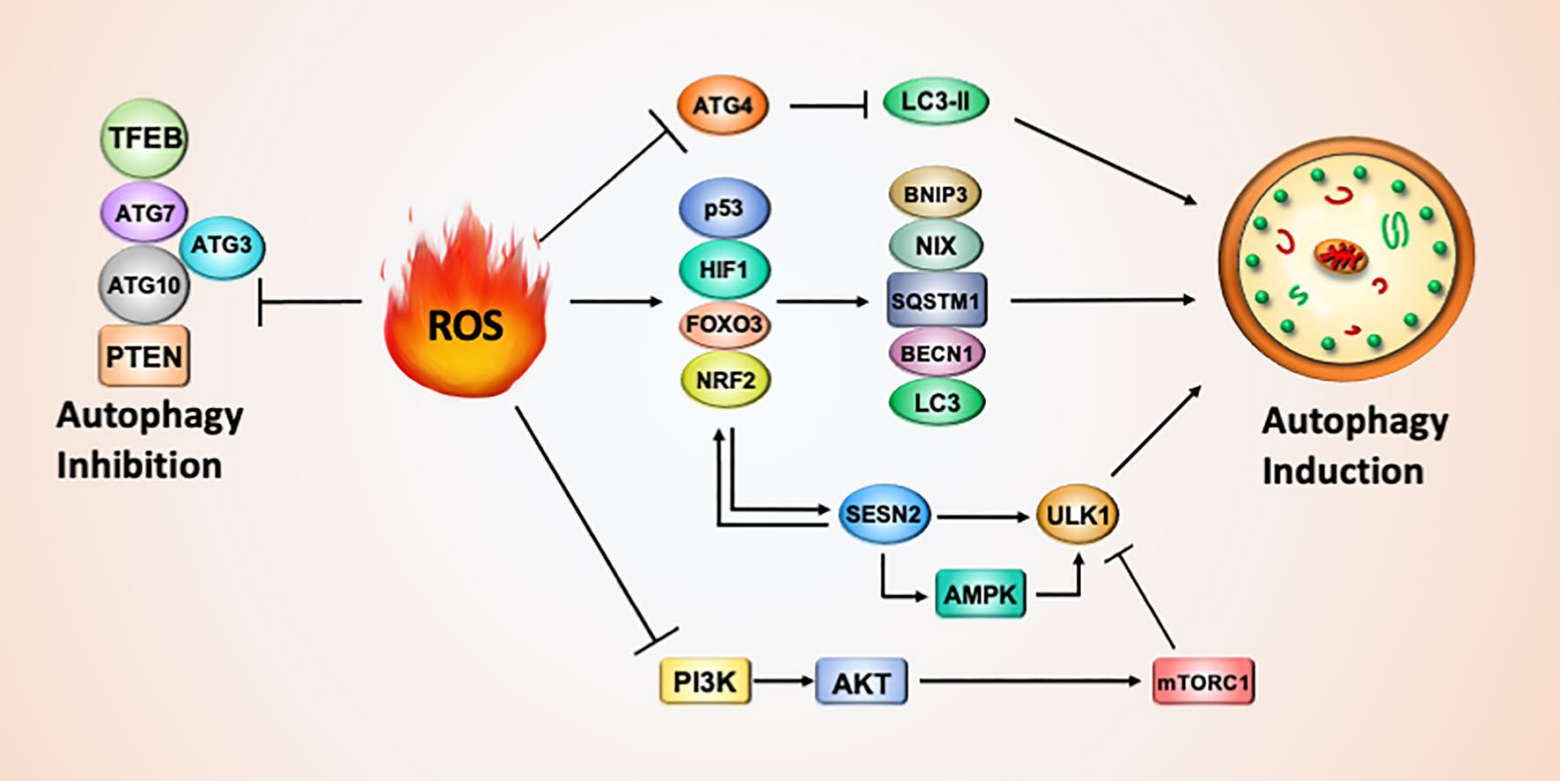
Dual role of ROS in autophagy induction and inhibition. ROS trigger the activation of transcription factors, such as p53, HIF1A and NRF2, to induce the expression of autophagy-related genes. ROS spatiotemporally oxidize and inactivate ATG4 to maintain lipidated LC3-II and autophagosome formation. ROS also block PI3K-AKT-MTORC1 signaling to initiate autophagy signaling. In contrast, ROS oxidize ATG proteins and PTEN to suppress autophagy

Autophagy can be induced by ROS through transcriptional (HIF-1*α*, NRF2, p53 and FOXO3) and posttranslational regulation (oxidation and phosphorylation) (Fig. 2).

ROS production has been reported to activate hypoxia-inducible factor-1*α* (HIF-1*α*), nuclear factor erythroid 2–related factor 2 (NRF2), p53 and forkhead box O-3 (FoxO3). These transcription factors drive the expression of the genes required for autophagy induction, including *BECN1, LC3, SQSTM1* and the mitophagy-associated genes *BNIP3* and *NIX* [56, 57].

Sestrins (SESNs) are another antioxidant cytoplasmic protein and consist of three members, SESN1, SESN2, and SESN3, in mammalian cells. ROS oxidize nucleic acids and cause DNA damage. The severe DNA damage may increase p53 transcriptional activity. The *SESN1* and *SESN2* genes are targets of p53; therefore, SESNs are induced in cells under oxidative stress [58]. Several other transcription factors are also reported to drive *SESNs* gene expression, such as NRF2 [59], HIF-1*α* [60], and the NH(2)-terminal kinase (JNK)/c-Jun pathway [61]. SESNs contain motifs required for the removal of ROS, including an N-terminal cysteine (C125) with an active site for oxidoreductase activity to reduce alkyl hydroperoxide radicals and a C-terminal aspartate-aspartate motif for mTORC1 suppression [62, 63]. Moreover, SESN2 interacts with KEAP1 to mediate its degradation with autophagy for further NRF2 activation and antioxidant gene expression, as mentioned above [64].

In terms of the effects of posttranslational modification of ROS on autophagy, SESN2 also binds to ULK1 and SQSTM1 to increase SQSTM1 phosphorylation at the UBA domain (S405/409), indicating that SESN2 recruits ULK1 to phosphorylate SQSTM1 and promotes autophagy [65]. In addition, SESN2 sustains AMPK activation to inhibit mTORC1 [66]. These observations provide links to the role of SESNs in autophagy in response to oxidative stress. In addition, starvation-induced H_2_O_2_ oxidizes ATG4 at Cys78 to spatiotemporally inactivate ATG4 and ensure that lipidated LC3-II can facilitate autophagosome formation before deconjugation [67]. In addition, ROS elevate AMPK phosphorylation and activity to inhibit mTORC1 [68]. Alternatively, ROS downregulate PI3K-AKT signaling to reduce mTORC1 activity for autophagy induction [69, 70]. As mentioned above, AMPK and mTORC1 are positive and negative regulators of ULK1, respectively. Thus, ROS can initiate autophagy through AMPK activation and mTORC1 inactivation.

#### ROS inhibit autophagy

In contrast, the autophagy core protein E1-like enzyme ATG7 and E2-like enzymes ATG10 and ATG3 consist of sulfhydryl groups, which are sensitive to ROS oxidation and inactivate enzyme activity (Fig. 2) [53]. The inactivation of these core enzymes of autophagy leads to autophagy reduction. ROS also inactivate PTEN, a phosphatase that negatively regulates PI3K-AKT-mTORC1, to diminish autophagy [54]. Moreover, Transcription Factor EB (TFEB) is a mater regulator to drive gene expression required for biogenesis of autophagosome and lysosome [71]. Low concentration (100 or 200 µM) of H_2_O_2_ activates TFEB and has no effects on cell viability, whereas high concentration (400 or 800 µM) of H_2_O_2_ inactivates TFEB and leads to neuron cell death [55]. Thus, ROS may initially oxidize and inactivate essential autophagy genes and then induce several pathways to reactivate autophagy and compensate for the redox status. Alternatively, ROS-modulated autophagy might rely on the context of cell types and the timing or conditions of stress for ROS generation.

### Regulation of ROS by autophagy

**Fig. 3:**
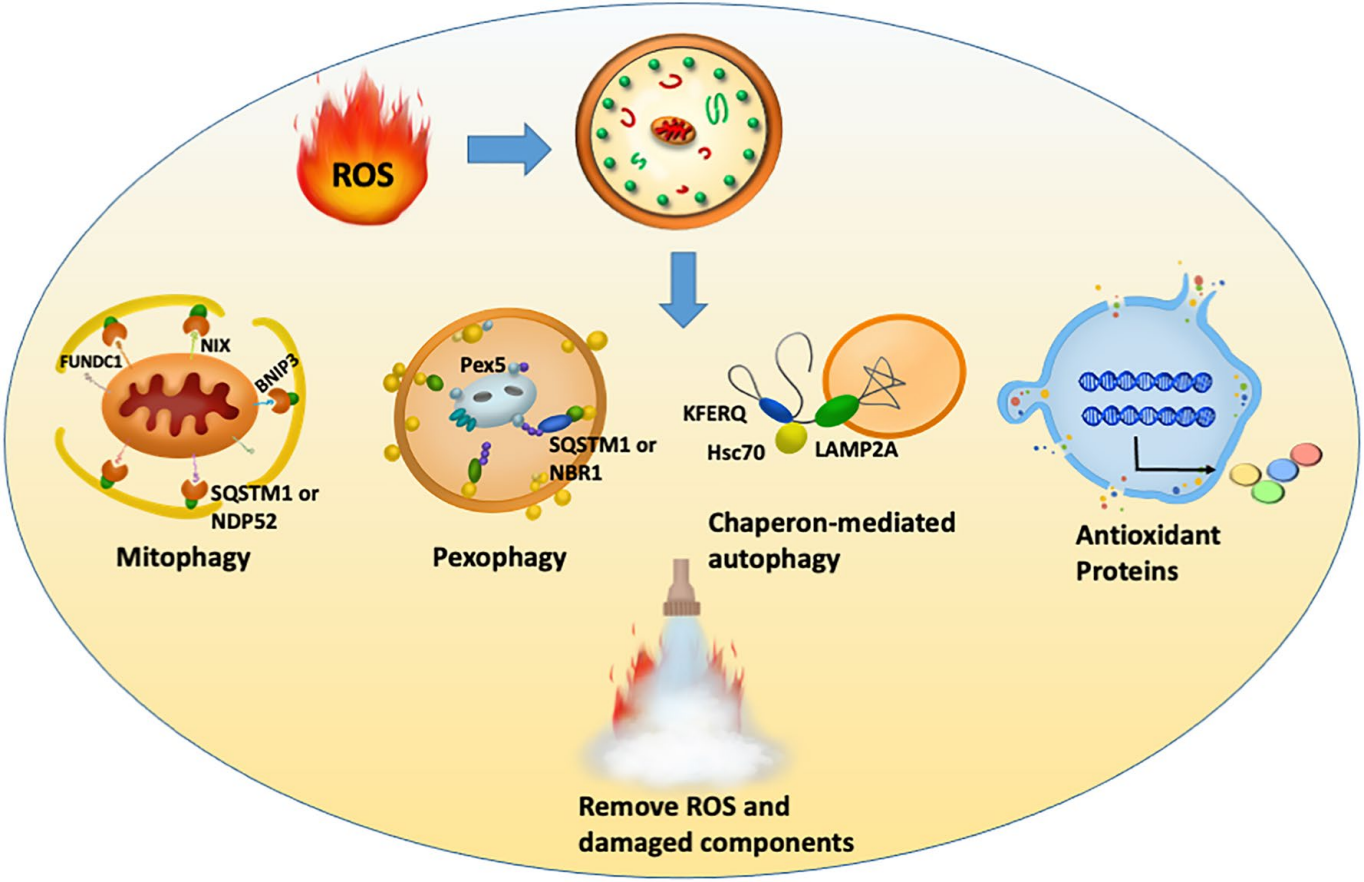
Pathways involved in ROS elimination by autophagy in cells under oxidative stress. Autophagy can degrade ROS-generating organelles, including mitochondria (mitophagy) and peroxisomes (pexophagy), by binding ubiquitinated proteins to autophagy receptors (SQSTM1, NBR1 and NDP52). Autophagy also removes unfolded proteins through chaperone-mediated autophagy. In addition, autophagy activates NRF2 to induce antioxidant gene expression to eliminate excessive ROS in cells

ROS stimulation accumulates impaired organelles and enhances cellular ROS in autophagy-deficient cells lacking BECN1, ATG5 or ATG7 [72, 73]. ROS-oxidized organelles and proteins can be removed by autophagy to protect cells [74, 75]. Autophagy is a form of quality control for cellular components, particularly for mitochondria, peroxisomes and proteins that are involved in ROS generation [10, 76]. Thus, although autophagy is modulated by ROS, autophagy also has a feedback loop to regulate ROS levels through transcription factor (NRF2, p53) activation or the degradation of damaged components, such as mitochondria, peroxisomes and unfolded proteins, as discussed below (Fig. 3).

#### Clearance of impaired organelles

Mitochondria and peroxisome are major ROS-producing organelles in cells. Mitophagy and pexophagy are types of selective autophagy to degrade impaired mitochondria and peroxisome, respectively. As mentioned above, cellular ROS are mainly produced from mitochondria, which are so-called mitoROS. MitoROS can be limited to regular oxidative phosphorylation reactions in the inner membrane of mitochondria in cells under normal conditions. In contrast, mitochondria also contain ROS scavenger systems, such as the superoxide dismutase (SOD) family of proteins [77], to convert O2^−^ into H_2_O_2_ to maintain redox homeostasis and GSH (glutathione) redox systems to decompose H_2_O_2_ into O_2_ and H_2_O [78]. Mitochondrial dysfunction leads to cellular ROS elevation [79]. Mitophagy is a selective autophagy pathway that degrades impaired mitochondria. Mitophagy defects result in the accumulation of impaired mitochondria and the elevation of cellular ROS and damage [80, 81]. Mitophagy is processed mainly via Parkin ubiquitination and BNIP3-NIX-FUNDC1 mitochondrial adaptor pathways.

Parkin is an E3 ubiquitin ligase and is phosphorylated at S65 by PTEN-putative kinase 1 (PINK1) [82]. The phosphorylation of Parkin is fully activated due to conformational changes to (i) eliminate autoinhibitory effects and (ii) bind charged E2 ligases [83]. Active Parkin ubiquitinates many mitochondrial proteins located in the outer membrane, matrix and inner membrane, such as voltage-dependent anion channel 1 and mitofusins (Mfn1 and Mfn2) [84, 85]. Ubiquitinated mitochondrial proteins associate with autophagy cargo receptors, such as SQSTM1, NDP52 and optineurin, to recruit damaged mitochondria to autophagosomes. The Rab signaling proteins RABGEF1, RAB5, and RAB7A, located on the mitochondrial surface, are also involved in the mitophagy recruitment process [86, 87]. Interestingly, Parkin translocates to mitochondria in cells under oxidative stress, indicating that Parkin is important for oxidative-stress-mediated mitophagy [88, 89].

Moreover, several other cargo adaptors are induced by oxidative stress to facilitate mitophagy, such as FUNDC1, NIX and BNIP3. These mitochondrial cargo adaptors include the LC3-interacting region, which connects mitochondria and autophagosomes to promote mitophagy [90]. FUNDC1-mediated mitophagy is positively and negatively regulated by ULK1 and Src kinase, respectively [90, 91].

Peroxisomes are organelles that undergo many metabolic pathways in cells, particularly pathways involved in lipid metabolism, such as the α- and β-oxidation of fatty acids, ketogenesis, and the metabolism of isoprenoids and cholesterol [92]. In addition to mitochondria, peroxisomes are another main organelle that produces intracellular ROS by releasing free electrons from several oxidases [93, 94]. Peroxisomes also contain many antioxidant enzymes to remove excessive ROS, including GPX, catalase, and SOD [95]. Defects or damage to peroxisomes may lead to intracellular ROS elevation, while damaged peroxisomes can be eliminated by pexophagy. Pexophagy starts with ataxia-telangiectasia mutated kinase (ATM) activation through ROS-mediated disulfide bond formation of ATM to dimerize and become its active form [96–98]. Active ATM promotes AMPK activation, which in turn phosphorylates ULK1 kinase for autophagy initiation [99, 100]. Additionally, ATM phosphorylates peroxisomal protein Pex5 at Ser141 to trigger Pex5 ubiquitination [101]. Ubiquitinated Pex5 then interacts with the autophagy receptors SQSTM1 and NBR1 to degrade damaged peroxisomes through pexophagy [7]. In addition, ROS are elevated in patients with ATM mutations and ATM-deficient mice [102, 103], supporting the notion that pexophagy can eliminate excessive ROS to maintain redox homeostasis and keep cells healthy.

#### Clearance of unfolded proteins by CMA

CMA is a specific type of autophagy that delivers unfolded proteins into lysosomes and degrades them in a chaperone-dependent manner. In contrast to proteasomal degradation, CMA requires a KFERQ pentapeptide sequence as a degradation signal in substrate proteins (approximately 30% soluble proteins) instead of ubiquitination [104]. When cells are under oxidative stress, proteins containing the pentapeptide sequence are unfolded to expose the sequence for binding with constitutive heat shock protein 70 (HSC70) [105]. A recent study also showed that some of pentapeptide non-existing proteins may create a KFERQ-like structure for HSC70 recognition in cells under oxidative stress [106]. The chaperone-associated complex is then translocated to lysosomes and imported by LAMP-2A for degradation. Moreover, *LAMP-2A* gene expression is induced in cells during oxidative stress [105, 107]. Silencing LAMP-2A impairs CMA and increases ROS-induced ferroptosis in retinal pigment epithelial ARPE-19 cells, while cysteine and glutamine supplementation rescue ROS-induced cell death [108]. Interestingly, increased macroautophagy is not able to restore ROS-induced damage in CMA-defective cells [109], suggesting that CMA is essential for cytoprotection in response to ROS.

#### Expression of antioxidant and autophagic pathways

**Fig. 4:**
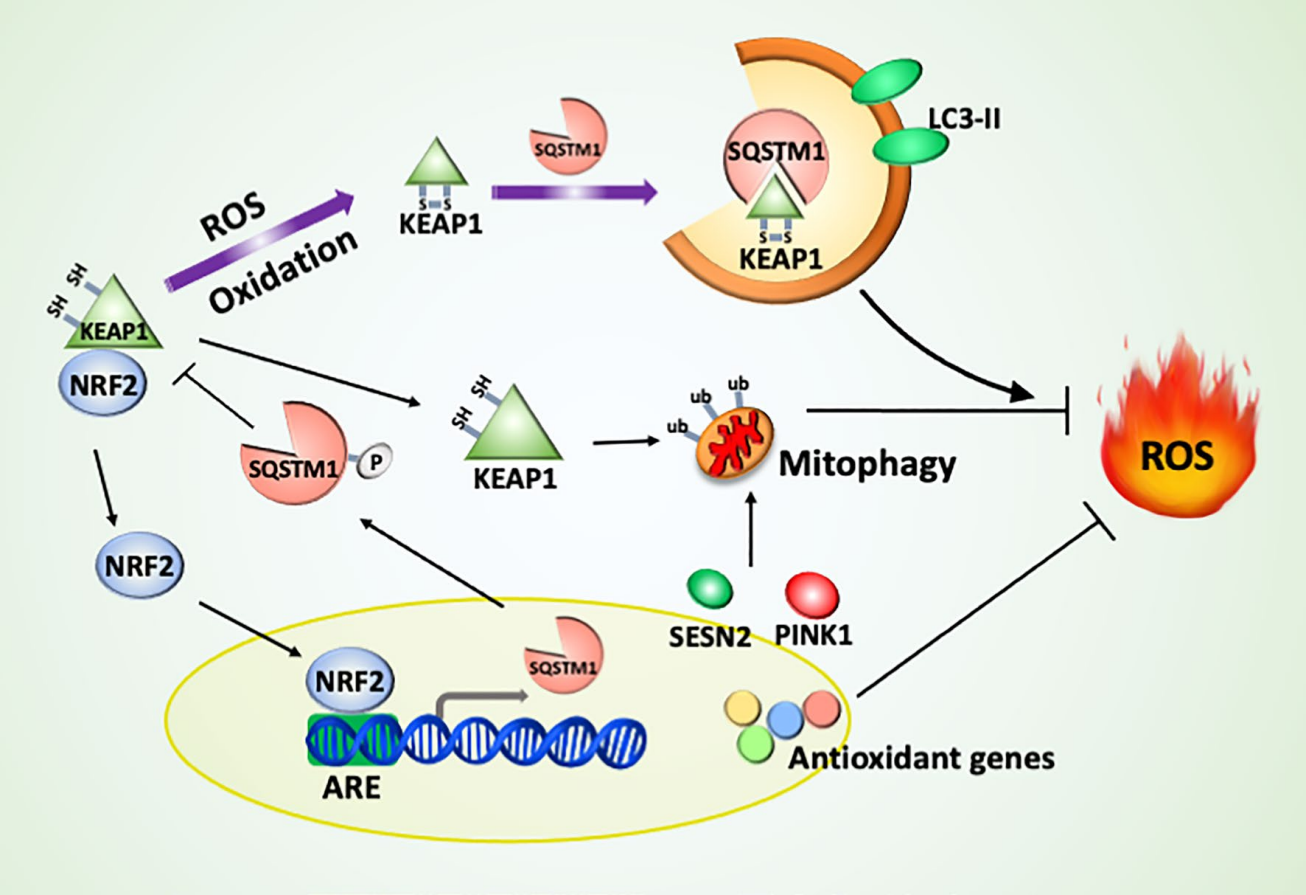
The mechanisms of NRF2 activation and antioxidation. KEAP1, an E3 ligase of NRF2, can be directly oxidized and recruited by SQSTM1 to autophagosomes for degradation. Liberated NRF2 can induce the gene expression of the antioxidant genes Sqstm1, Sesn2 and Pink. The induced SQSTM1 is phosphorylated to interrupt the binding between KEAP1 and NRF2 for further positive feedback activation of NRF2. The released KEAP1 and induced SESN2 and PINK promote mitophagy to remove damaged mitochondria

NRF2 is the major transcription factor involved in autophagy-mediated antioxidant mechanisms. NRF2 is normally ubiquitinated by the E3 ligase Kelch-like ECH-associated protein 1 (KEAP1) and results in degradation [110]. KEAP1 can be eliminated by autophagy, specifically through interruption by SQSTM1 (Fig. 4).

Cysteine residues of KEAP1 are oxidized to form disulfide bonds and lead to conformational changes to release NRF2 [111]. NRF2 can enter the nucleus and bind to the promoter with an antioxidant-response element (ARE, 5′-TGACXXXGC-3′) to turn on the expression of several antioxidant, detoxification enzymes and autophagy genes, including NADPH quinone dehydrogenase 1 (*NQO1*), glutathione S-transferase (*GST*) genes and *SQSTM1* [110, 112–117]. SQSTM1 is phosphorylated by mTORC1 to compete for the interaction between KEAP1 and NRF2, thereby preventing NRF2 degradation [118, 119]. ATG8-defective mice accumulate SQSTM1, resulting in the hyperactivation of NRF2 and limiting oxidative stress [119], whereas Nrf2-knockout mice exhibit elevated oxidative stress [120]. Thus, NRF2 and SQSTM1 are parts of a positive feedback loop to reduce oxidative stress. Moreover, NRF2 induces the gene expression of *SESN2* and *PINK1* to promote macroautophagy and mitophagy in the cell response to oxidative stress, respectively [121, 122]. In addition to the Parkin E3 ubiquitin ligase, SQSTM1 triggers the translocation of KEAP1, an E3 ubiquitin ligase, to mitochondria for mitophagy activation [123].

### The effects of ROS-mediated autophagy on survival and death

Autophagy acts as a recycling pathway to eliminate impaired proteins, organelles or pathogens to maintain cell health. Intracellular oxidative stress significantly regulates autophagy. In addition, autophagy regulates ROS levels in cells via mitophagy, pexophagy, proteasomal, and CMA pathways. Furthermore, autophagy can directly regulate antioxidant pathways (i.e., NRF2 and SESN molecules) to modulate redox homeostasis and cell survival. Thus, autophagy is thought to be a cytoprotective mechanism in cells under starvation or stressed conditions. However, excessive stress-induced autophagy may lead to cell death, which is called autophagic cell death [124]. Autophagic cell death meets the criteria that i) autophagic flux is increased and ii) the ablation of autophagy inhibits cell death to ensure that cell death is caused by autophagy rather than dying cells with protective autophagy. Autophagic cell death is observed under certain stresses, particularly oxidative stress. Hydrogen peroxide (H_2_O_2_) exposure or reactive oxygen species (ROS) generation through the disruption of mitochondrial function induces autophagic cell death [125, 126]. Genetic or pharmacological ablation of autophagy diminishes cell death, whereas the apoptosis inhibitor Z-VAD has no effects on cell death. The mechanisms of autophagic cell death can depend on certain cells in response to different conditions. For example, glycogen synthase kinase 3-beta, ryanodine receptor 3, and PARKIN are involved in mitophagy and autophagic cell death in hippocampal neural stem cells during insulin withdrawal [127]. Notably, the ryanodine receptor, which controls calcium release from the ER, is activated and leads to autophagic cell death in a variety of apoptosis-resistant cancer cells when exposed to neferine [128]. These observations suggest that excess autophagy may require lots of autophagy components, such as lipids, ATG proteins and signaling factors, and cause cellular burden/stress and death.

## Autophagy and oxidative stress in the pathogenesis of retinal diseases

**Fig. 5:**
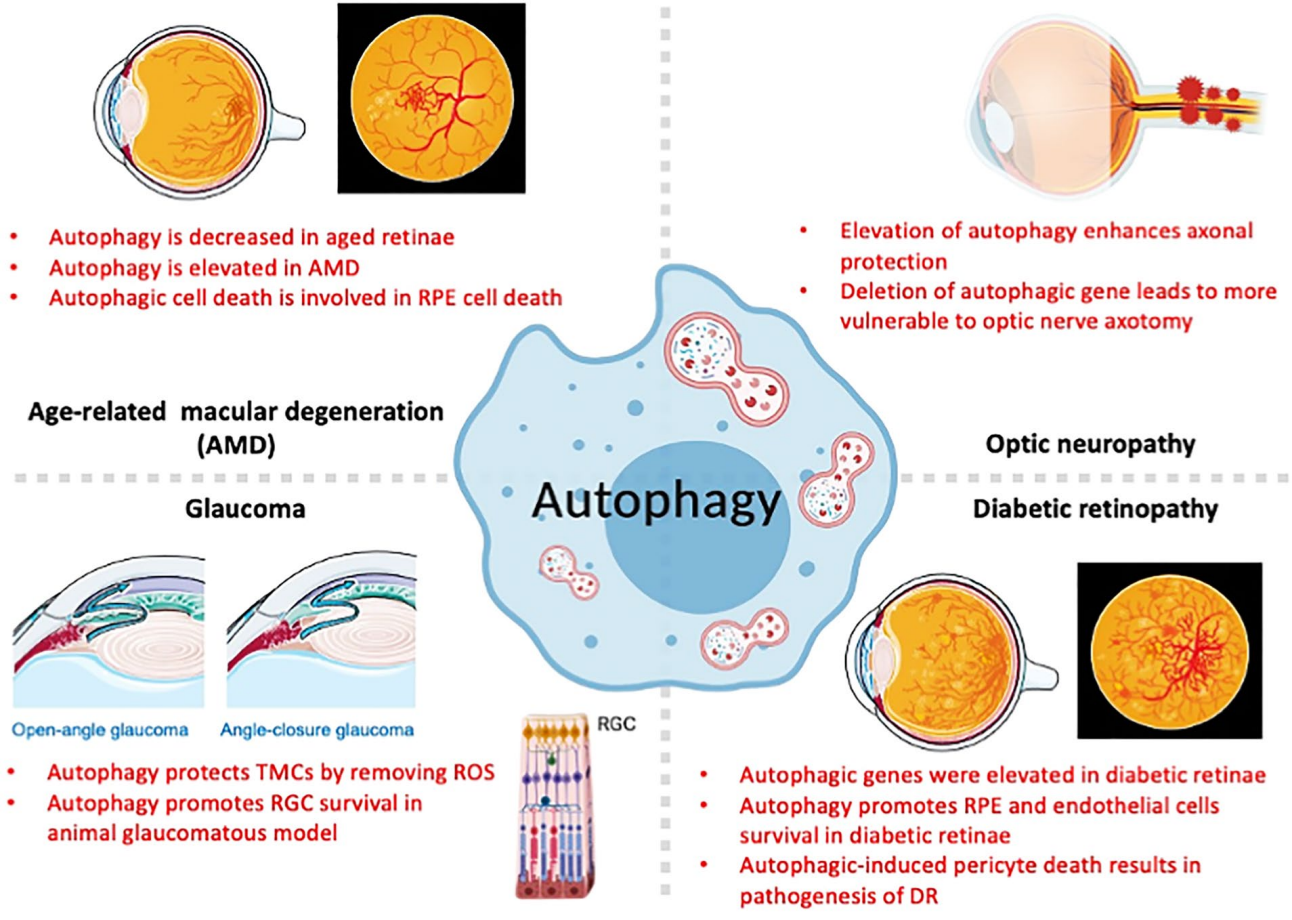
The potential involvement of autophagy in different ocular diseases. Autophagy is involved in several ocular diseases, including age-related macular degeneration (AMD), glaucoma, optic neuropathy, and diabetic retinopathy (DR). In general, autophagic flux protects retinal cells from oxidative-induced insult. However, excess autophagic flux may cause cell death and lead to retinal degeneration, such as in cells of the retinal pigment epithelium (RPE) in AMD and pericytes in DR. This figure was created partially with BioRender.com and Smart.Servier.com

The roles of autophagy and oxidative stress in different ocular diseases are shown as schematic diagram (Fig. 5) and described below:

### Glaucoma and optic neuropathies

Glaucoma is the second leading cause of blindness worldwide and was estimated to affect ~ 80 million people in 2020 [129]. Retinal ganglion cell (RGC) loss and extensive axon degeneration are the main signs of glaucoma. The elevation of intraocular pressure (IOP), one of the main causes of glaucoma, induces axonal degradation and RGC death, and this phenomenon is exacerbated with aging [130]. RGCs have long axons with a high density of mitochondria, which makes them more sensitive to oxidative stress [131]. In addition, growing evidence indicates that reactive oxygen species (ROS) play a key role in the pathogenesis of primary open angle glaucoma (POAG) by attacking trabecular meshwork (TM) cells [132, 133]. A recent study demonstrated that autophagy activation can be triggered by IOP elevation and mechanical stretch in TM cells, and primary cilia are critical for IOP homeostasis and autophagy activation [134].

In the optic nerve, oxidative stress is elevated after nerve crush injury [135], which triggers autophagy in RGCs, Müller cells [131, 136] and the primary visual cortex [137]. In addition, retinal hypoxia and axonal damage of the optic nerve also induce autophagy [138, 139]. Pharmacological induction of autophagy by rapamycin promotes RGC survival and after optic nerve axotomy in a mouse model [140]. The dysregulation of autophagy contributes to neurodegeneration in glaucoma [141]. SIRT1 activation enhances axonal protection in TNF-α-induced optic nerve degradation by elevating the autophagy pathway [142]. In addition, BNIP3L-mediated mitophagy is required for optic nerve oligodendrocyte differentiation [143]. Autophagy is enhanced after optic nerve crush (ONC) damage in zebrafish RGC axons, somas, and growth cones [144].

In a transgenic animal model, *Atg4b*-/- mice were more susceptible to stress, as optic nerve axotomy resulted in reduced RGC survival in these animals compared with WT mice, suggesting that autophagy levels can alter the capability of RGCs to respond to axonal stress [145]. In a mouse model, retinae with *Atg5* deletion by intravitreally injected with adeno-associated virus (AAV)2-ATG5^fl/fl^ were more vulnerable to optic nerve axotomy than control mice [140]. Cells from autophagy-deficient animals show increased levels of ROS [131]. In addition, mutations of optineurin (OPTN) were known to associated with normal tension glaucoma [146]. Genetic mutation of *OPTN* at residue E50K was reported to affect autophagy and cause the apoptosis of RGCs. The disruption of OPTN^E50K^ induced autophagy affected the degradation of TDP-43, which led to glaucomatous retinal neurodegeneration [147].

MTOR, the mammalian target of rapamycin, plays an important role in RGCs and glial cells for retinal development and axonal survival after ON injury [148]. Many studies have reported that the mTOR inhibitor rapamycin is used to induce autophagy and treat glaucoma in rodent models [149], showing the promotion of RGC survival in the ischemia/reperfusion injury model caused by IOP elevation [150]. Autophagy decreases with aging in the retina, and the induction of autophagy shows neuroprotective effects in a glaucoma animal model [151]. Although most evidence shows that autophagy is protective in glaucoma, an opposing result showed that the inhibition of autophagy by 3-methyladenine (MA) alleviates acute axonal degeneration in a rat model [139]. Another study in a zebrafish model showed that the inhibition of autophagy promotes axonal regrowth [144].

### Age-related macular degeneration

There are two main types of age-related macular degeneration (AMD), dry AMD (geographic atrophy) and wet AMD (choroidal neovascularization, CNV). In dry AMD, patients develop yellow deposits, called drusen, in their macula. Without appropriate treatment, drusen become increasingly numerous, causing light-sensitive cell death in the macula and leading to multiple blind spots in the central vision. Once AMD reaches an advanced stage, blood vessels grow from underneath the macula and leak blood and fluid into the retina, eventually forming a scar and leading to permanent loss of central vision [152]. Among various factors, oxidative stress is strongly implicated in AMD [153, 154]. Aging [155] and oxidative stress [156] were reported to be involved in retinal neovascularization, which is an advanced progression of AMD. However, a study reported that oxidative stress-induced nuclear factor kappa B (NF-κB) signaling promotes retinal pigmented epithelial (RPE) cell survival through increased autophagy [157]. In addition, the catalytic subunit of human telomerase (hTERT) is known to be associated with AMD by interacting with mTORC1 (mechanistic target of rapamycin complex 1) and PINK1 (PTEN-induced kinase 1), which activates macroautophagy and mitophagy, respectively [158]. A decrease in autophagic activity with age observed in many tissues has been proposed to contribute to the aggravation of age-related diseases [159].

The RPE is responsible for the phagocytosis of photoreceptor outer segments (POSs) [155] and is considered one of the most important retinal cells involved in AMD [160]. With increasing age, lipofuscin accumulates in the RPE and contributes to the pathogenesis of AMD [155]. Autophagy regulates the death of RPE cells in AMD [161]. Impairing autophagy in RPE leads to inflammasome activation and enhances macrophage-mediated angiogenesis. In vitro inhibition of rotenone-induced autophagy in RPE cells elicits caspase-3-mediated cell death [162]. The autophagy marker ATG5 was observed in the drusen of human normal old eyes and was even more in AMD eyes, suggesting that autophagy contributes to the formation of drusen in aged RPE [163]. The deletion of ATG5 leads to apoptosis in the outer nuclear layer (ONL) of the mouse retina [164]. Compared with normal eyes, the RPE from human donor AMD eyes shows more autophagosome expression and is more susceptible to oxidative stress [165], which suggests that dysfunctional autophagy contributes to the pathophysiology of AMD [166]. The dying RPE triggered by autophagic pathway would be engulfed by human macrophages and dendritic cells (DCs), and a failure of engulfment in the retina may result in the accumulation of debris and the progression of AMD [167]. Oxidative stress was reported to induces autophagy and cell death in RPE cells [167, 168]. Silencing autophagy essential genes (*ATG5/ATG7*) diminishes cell death in ARPE-19 cells treated with H_2_O_2_ [169], suggesting that autophagic cell death is involved in RPE cell death when cells are exposed to excessive oxidative stress.

### Diabetic retinopathy

Diabetic retinopathy (DR) is a severe ocular complication of diabetes and accounts for ~ 5% of all cases of blindness worldwide. Hyperglycemia, the common symptom of diabetes, is known to induce oxidative stress in retinal cells [170]. The early stage of DR is usually termed nonproliferative diabetic retinopathy (NPDR). In NPDR, the blood vessels in the retina close off, and blood cannot reach the macula, which is also called macular ischemia. DR that progresses to an advanced stage is termed proliferative diabetic retinopathy (PDR). In eyes with PDR, the retina begins to grow new blood vessels, called neovascularization. These new vessels often cause blood leakage into the vitreous and block vision. To test the correlation between diabetes and autophagy, a study conducted using a RPE cell culture exposed to hyperglycemic conditions showed that high glucose (HG) induces the autophagosome formation regulated by ROS-mediated ER stress signaling [171]. In a diabetic mouse model, autophagosome and autophagic proteins (Beclin-1 and Atg5) were elevated in the diabetic retina, leading to a loss of rod photoreceptors and a reduction in the thickness of the outer and inner synaptic layers [172]. In addition, HG promotes advanced glycation end product (AGE) formation, causing oxidative stress and inflammatory responses that alter vascular function in the diabetic retina, resulting in diabetic complications [173, 174]. Strong evidence indicates that autophagy plays a protective role in suppressing inflammasome activation [175]. On the other hand, an increase in autophagy through the inhibition of mTOR signaling promotes endothelial cell survival in diabetic retinas, which can alleviate the progression of DR [176]. Conversely, a long-term increase in autophagy induces pericyte cell death, which may result in the pathogenesis of DR [177].

## Current therapy for retinal degeneration

### Glaucoma

IOP elevation is one of the main causes of glaucoma. In the clinic, glaucoma is often treated with prescription eyedrops. A variety of eyedrops are used, including prostaglandins [178], beta blockers [179], alpha-adrenergic agonists [180], carbonic anhydrase inhibitors [181], Rho kinase inhibitors [182] and cholinergic agents [183], all of which regulate glaucoma through different molecular mechanisms. These eyedrops function to decrease eye pressure by improving the drainage of fluid from the eye or by decreasing the amount of fluid that the eye makes. However, some patients complain of side effects unrelated to the eyes due to the molecular absorption of eyedrops into the bloodstream.

In certain patients with advanced glaucoma, eyedrops fail to reduce eye pressure to the desired level, whilst patients with acute angle-closure glaucoma require surgical procedures or alternative treatments, including laser therapy [184], filtering surgery [185], drainage tubes [186], or minimally invasive glaucoma surgery [187].

RGCs degenerate in glaucoma, which leads to permanent vision loss. Thus, a cell replacement strategy was considered a potential therapy to treat RGC loss. In the past decade, scientists have been able to differentiate human stem cells into RGC-like cells [188–192]. However, how to scale up donor cells, promote long-term cell survival and enhance synaptic integration into the visual circuit remains a challenge for stem cell therapy [193].

### Optic neuropathies

Optic neuropathies take various forms, including nonarteritic anterior ischemic optic neuropathy (NAION), which damages the optic nerve and results from a change in blood flow or optic nerve trauma due to acute injury to the optic nerve. However, to date, there is no effective treatment for NAION. To slow the progression of NAION, treatment focuses on controlling blood pressure, reducing the symptoms and preventing NAION from damaging the other eye.

On the other hand, arteritic ischemic optic neuropathy treatment also aims to prevent further damage to the other eye and typically involves the use of anti-inflammatory drugs. Treatment depends entirely on the underlying condition or problem that causes the neuropathy and requires a full evaluation from an eye specialist.

Glaucoma is another main cause of optic neuropathy. Axons of RGCs degenerate in optic nerve injury and do not regrow; thus, what regulates axon regeneration remains a field of interest to scientists. Many studies have shown the promotion of axon regeneration by molecular therapies [194–198]. However, the length of regenerative axons and synaptic reconnection are still limited [199].

Optic glioma, which usually occurs in childhood, also leads to optic neuropathy and vision loss [200, 201]. A recent study indicated that light plays an important role in glioma formation during eye development [202]. Since light exposure induces photooxidative stress [203], which could induce autophagy, it would be interesting to ask whether autophagy regulates the formation of glioma-induced optic neuropathy in future studies.

### Age-related macular degeneration

To date, there is no cure for macular degeneration. However, several treatments, mainly anti-vascular endothelial growth factor A (VEGFA) class, may slow the progression of AMD or maintain existing vision. For example, the anti-angiogenesis drugs aflibercept (Eylea) [204] and bevacizumab (Avastin) [205] are used to block the creation of blood vessels and the subsequent leakage from these vessels that cause wet macular degeneration. A portion of the lost vision of many AMD patients who have taken these drugs has been improved [206]. If AMD is advanced, the patient might need to receive this treatment multiple times and such treatment is applied only in advanced AMD, which requires multiple injections.

In some patients, ophthalmologists recommend performing laser therapy by applying high-energy laser light to destroy abnormal blood vessels growing in the eye [207]. Alternatively, the doctor may perform photodynamic laser therapy by injecting the light-sensitive drug verteporfin (Visudyne) into the bloodstream, which is absorbed by abnormal blood vessels [208]. In addition, there are adjuvant devices such as special lenses or electronic systems for creating larger images of nearby things, which can help those who have vision loss due to macular degeneration and maximize their remaining vision [209].

### Diabetic retinopathy

Neovascularization is the hallmark of DR. In the clinic, intravitreal injection of anti- VEGF agents such as bevacizumab, aflibercept, ranibizumab [210], which we described above in the AMD section, is the primary procedure to slow the progression of DR. To reduce the swelling of the retina, scatter laser surgery might be used to help block leaking blood vessels. In addition, laser surgery also helps shrink blood vessels and prevent them from proliferation. However, laser treatment is associated with a risk of peripheral (side), color, and night vision loss.

Once advanced proliferative DR (PDR) develops, an ophthalmologist may recommend an alternative surgery called vitrectomy [211], a procedure to remove vitreous gel containing blood from leaking vessels and scar tissue in the back of your eye. However, the procedure is associated with some risks, including ocular infection, cataract formation and retinal detachment.

In the development of a less invasive treatment, pharmaceutical DR treatment strategies have been explored in recent decades. Aldose reductase (AR), the enzyme that converts glucose to sorbitol, is involved in a variety of diabetic complications, including DR [212]. Many AR inhibitors have been developed to alleviate the progression of diabetic complications and ocular inflammation in animal models [213]. However, renal and liver toxicity remains a concern in clinical trials [214, 215].

### Role of autophagy in current ocular degeneration therapy

In addition to operating the procedures or surgeries mentioned above, slowing the onset or progression of such diseases still a goal for ocular degeneration therapy. Since ROS is one of the main causes of many degenerative diseases in the eye and autophagic pathway could clean the cells of all irreversibly oxidized biomolecules [74], developing topical drug based on mediating autophagic pathway would be a great of interest for scientists and clinicians. Many autophagic inducers were tested in animal models or have been used in the clinic. We next explore the detail of autophagic inducers or inhibitors in the next chapter.

## Effects of autophagic inducers and inhibitors on retinal degenerative diseases

### Steroids

In a rat glaucoma model, neurosteroids induced the autophagy pathway to protect retinal neurons via GABRs/GABAA receptors [216]. However, another study reported that steroid therapy in the eye leads to the dysregulation of TMCs and glaucoma pathologies by inhibiting the autophagosome biogenesis pathway [217]. More studies are needed to conclude the effect of steroids on retinal neurons.

### Rho kinase inhibitor

Ripasudil is a rho-associated coiled-coil-containing protein kinase 1 (ROCK1) inhibitor. In the clinic, ripasudil is a key component in ophthalmic solutions for treating glaucoma by reducing IOP [218]. In a rodent model study, ripasudil was shown to enhance intraaxonal autophagy and promote axonal protection [219].

### mTORC1 inhibitors

#### Rapamycin

The activation of autophagy, modulated by the rapamycin-induced inhibition of mTORC1 signaling, is able to prevent the harmful AMD-related aging of RPE cells [220]. The mTOR inhibitor rapamycin ameliorates the high glucose-induced inflammatory responses and ROS in the RPE [221]. Rapamycin plays a protective role in a rodent chronic hypertensive glaucoma model [222] and significantly increases RGC survival following optic nerve transection [140]. The heteroplasmic mtDNA G11778A mutation is the most common cause of Leber's hereditary optic neuropathy. An in vitro study showed that rapamycin treatment induces the colocalization of mitochondria with autophagosomes, resulting in less damage from the G11778A mutation [223]. The findings of this study suggest the potential of rapamycin as a therapeutic strategy to treat Leber's hereditary optic neuropathy.

#### Everolimus

Fibroblast-mediated scar formation is a common complication of glaucoma filtering surgery. A study showed that everolimus, another mTORC1 inhibitor, suppresses the proliferation of fibroblasts in the eye after surgery [224]. In addition, everolimus has been shown to suppress angiogenesis [225], which is the onset of wet AMD [226] and DR [227]. Everolimus is also a common therapy for kidney transplant recipients at a late post-transplant stage [228]. However, a clinical case study reported that long-term administration of immunosuppressant everolimus or tacrolimus (an analog of everolimus) in a transplant recipient might be a risk factor for the development of posterior reversible encephalopathy syndrome or optic neuropathy [229, 230].

#### Temsirolimus

Temsirolimus, an analog of everolimus, inhibits RPE and endothelial cell proliferation and migration, and decreases VEGF and PDGF expression [231], which can be used to alleviate AMD and DR. In addition, sirolimus is also considered an antiangiogenic drug for DR progression [232].

### AMPK activator

Metformin is able to trigger autophagy through AMPK activation and the subsequent inhibition of mTORC1 signaling [233]. Metformin is used to control blood sugar and is considered to reduce the risk of the onset of AMD [234], glaucoma [235] and DR [236] in diabetic patients.

### mTOR-independent autophagy inducer

Lithium (LiCl) induces autophagy through an mTOR-independent pathway [237]. In animal studies, LiCl was reported to be an autophagy inducer for alleviating the progression of glaucoma [238], DR [239] and optic neuropathy [240].

### Inhibitors of autophagosomes and lysosomes

Chloroquine (CQ) and hydroxychloroquine (HCQ) are autophagic inhibitors popularly used as antitumor agents [241]. Both CQ and HCQ have been reported to cause RGC damage [242, 243]. In the clinic, the mean values of quantified fundus autofluorescence (QAF, an indirect approach to measuring lipofuscin in the RPE in vivo) were significantly higher in patients receiving CQ/HCQ than in healthy controls, indicating that CQ/HCQ treatment leads to retinal damage [244]. Another case study reported the incidence of blindness in a population of rheumatic patients treated with HCQ [245]. In addition, CQ completely abolished the antiapoptotic effect of the somatostatin analog octreotide in hyperglycemia-treated retinal tissue [246], suggesting that CQ might worsen the progression of DR.

On the basis of the literature reviewed above, the promotion of the autophagic pathway plays a protective role in retinal degenerative diseases. The application of autophagic inhibitors in the clinic requires more research and assessments of the risk of their unfavorable side effects, especially in the eyes.

## Conclusion

ROS-mediated damage to cellular components is highly associated with the pathogenesis of several ocular diseases, as mentioned above. Autophagy is one of the main routes to eliminate damaged components in cells in response to oxidative stresses. ROS may initially oxidize several enzymes, including ATG proteins, to inhibit autophagy. ROS then trigger signaling pathways to activate autophagy to form a negative feedback loop to suppress ROS. Though the role of autophagy in the pathogenesis of ocular diseases might vary, autophagy should be a beneficial pathway for ocular cell survival under short-term oxidative stress. As aforementioned (Table 2), several autophagy inducers, particularly the AMPK inducer and mTORC1 inhibitors, have been shown to diminish the severity of ocular diseases in preclinical and clinical studies. In contrast, autophagy inhibitors CQ or HCQ are harmful in ocular diseases. Additionally, Neurofibromatosis 1 (NF1) mutation was reported to develop optic pathway gliomas [202], which leads to permeant blindness. Studies showed that activation of the mTOR pathway has been identified in benign and malignant NF1 tumors [247, 248], suggesting that activation of autophagy by inhibiting mTOR pathway could be a potential therapeutic strategy for optic neuropathy in patients with glioblastoma. However, metformin inhibits mitochondrial enzymes to activate AMPK, and the effects on cell protection could be AMPK- or autophagy-dependent and autophagy-independent [249, 250]. mTORC1 not only regulates autophagy signaling but also modulates cell differentiation, cell proliferation, angiogenesis and inflammation [251]. Therefore, more research on the role of autophagy in ocular diseases is required, particularly in clinical settings. The limitations of research on the role of autophagy in clinical ocular diseases are mainly due to the following: (i) the ocular structure of animals cannot completely reflect that in patients, (ii) a precise assay for autophagic flux in patients is lacking, (iii) specific autophagy modulators as clinical drugs are lacking, and (iv) the role of autophagy in different ocular disease types and stages might vary. Nevertheless, this review sheds light on autophagy modulation as an intervention for ocular diseases.

**Table 2:** Effects of FDA-approved autophagy-target drugs on retinal degenerative diseases

Drug	Mechanism	Role	Diseases	Physiologic effects	References
Chloroquine (CQ) & hydroxychloroquine (HCQ)	Autophagy inhibition to Autophagosome & Lysosome	Harmful	Glaucoma	Treatment of CQ and HCQ causes RGC and retinal damage	[244–246]
			Diabetic retinopathy	CQ worsens the progression of diabetic retinopathy	[248]
			Blindness	Rheumatic patients treated with HCQ leads to blindness	[247]
Rapamycin	Autophagy activation by mTORC1 inhibition	Protective	Glaucoma	Rapamycin is neuroprotective in a chronic hypertensive glaucoma model and increases RGC survival following optic nerve transection	[142, 224]
			AMD	Rapamycin prevents AMD-related aging of RPE cells	[222]
			Diabetic retinopathy	Rapamycin ameliorates the high glucose-induced ROC in the RPE	[223]
			Optic neuropathy	Rapamycin-induced autophagy results in less damage from G11778A mutation, the most common cause of Leber’s hereditary optic neuropathy	[225]
Everolimus	Autophagy activation by mTORC1 inhibition	Protective	Glaucoma	Everolimus suppresses the scar formation in glaucoma filtering surgery in an animal model	[226]
			AMD	Everolimus suppresses angiogenesis molecular pathways in the onset of wet AMD	[228]
			Diabetic retinopathy	Everolimus suppresses angiogenesis molecular pathways in the onset of diabetic retinopathy	[229]
		Harmful	Optic neuropathy	Long-term administration of everolimus may cause reversible encephalopathy syndrome and bilateral optic neuropathy after kidney transplantation	[231, 232]
Temsirolimus	Autophagy activation by mTORC1 inhibition	Protective	AMD	Temsirolimus inhibits RPE and endothelial cell proliferation and decreases VEGF and PDGF expression	[233]
			Diabetic retinopathy	Temsirolimus is considered as an antiangiogenic drug for diabetic retinopathy progression	[234]
Metformin	Autophagy activation by AMPK activation and subsequent inhibition of mTORC1 signaling	Protective	Glaucoma	Metformin is used to control blood sugar and is considered to reduce the risk of the onset of glaucoma, AMD, and diabetic retinopathy in diabetic patients	[236–238]
			AMD		
			Diabetic retinopathy		
Lithium (LiCl)	Autophagy activation by mTOR-independent pathway	Protective	Glaucoma	In animal studies, LiCl was reported as an autophagy inducer, which could alleviate the progression of glaucoma, diabetic retinopathy, and optic neuropathy	[240–242]
			Diabetic retinopathy		
			Optic neuropathy		
Ripasudil	Autophagy activation by inhibition of rho-associated coiled-coil containing protein kinase 1 (ROCK1)	Protective	Glaucoma	Ripasudil is the key component in ophthalmic solutions for treating glaucoma by reducing IOP Ripasudil promotes axonal protection in an animal model	[220, 221]
Steroids	Autophagy activation by GABA_A_ receptor	Protective	Retinal degeneration	Neurosteroids induces the autophagy pathway to protect retinal neurons	[218]
	Inhibiting autophagosome biogenesis pathway	Harmful	Glaucoma	Steroid therapy in the eye leads to the dysregulation of TMCs and develop glaucoma pathology	[219]

RGC: Retinal ganglion cell; AMD: age-related macular degeneration; mTORC1: mammalian target of rapamycin complex 1; RPE: retinal pigment epithelium; VEGF: vascular endothelial growth factor; PDGF: platelet-derived growth factor; AMPK: AMP-activated protein kinase; IOP: intraocular pressure; TMC: trabecular meshwork cell

## Data Availability

Not applicable.

## Abbreviations

AGE: Advanced glycation end product
AMD: Age-related macular degeneration
AR: Aldose reductase
ATM: Ataxia-telangiectasia mutated
CMA: Chaperone-mediated autophagy
FoxO3: Forkhead box O-3
HIF-1*α*: Hypoxia-inducible factor-1*α*
HOPS: Homotypic fusion and protein sorting
IOP: Intraocular pressure
KEAP1: Kelch-like ECH-associated protein 1
LIR: LC3-interacting region
mTORC1: Mechanistic target of rapamycin complex 1
NPDR: Nonproliferative diabetic retinopathy
NQO1: NADPH quinone dehydrogenase 1
NRF2: Nuclear factor erythroid 2–related factor 2
ONC: Optic nerve crush
ONL: Outer nuclear layer
PAS: Pre-autophagosomal structure
PDR: Proliferative diabetic retinopathy
PI3K: Phosphatidylinositol 3 kinase
PI3P: Phosphatidylinositol 3-phosphate
PINK1: PTEN-putative kinase 1
POAG: Primary open angle glaucoma
POSs: Photoreceptor outer segments
RGC: Retinal ganglion cell
RPE: Retinal pigmented epithelial
ROCK1: Rho-associated coiled-coil-containing protein kinase 1
ROS: Reactive oxygen species
SOD: Superoxide dismutase
TFEB: Transcription Factor EB
TM: Trabecular meshwork
UBA: Ubiquitin-associated
UVRAG: UV radiation resistance-associated
VEGFA: Vascular endothelial growth factor A

## References

[1] De Duve C, Wattiaux R (1966). Functions of lysosomes. Annu Rev Physiol.

[2] Kawamata T, Kamada Y, Kabeya Y, Sekito T, Ohsumi Y (2008). Organization of the pre-autophagosomal structure responsible for autophagosome formation. Mol Biol Cell.

[3] Matsuura A, Tsukada M, Wada Y, Ohsumi Y (1997). Apg1p, a novel protein kinase required for the autophagic process in *Saccharomyces cerevisiae*. Gene.

[4] Clark SL (1957). Cellular differentiation in the kidneys of newborn mice studies with the electron microscope. J Biophys Biochem Cytol.

[5] Novikoff AB (1959). The proximal tubule cell in experimental hydronephrosis. J Biophys Biochem Cytol.

[6] Ohsumi Y (2014). Historical landmarks of autophagy research. Cell Res.

[7] Rogov V, Dotsch V, Johansen T, Kirkin V (2014). Interactions between autophagy receptors and ubiquitin-like proteins form the molecular basis for selective autophagy. Mol Cell.

[8] Anding AL, Baehrecke EH (2017). Cleaning house: selective autophagy of organelles. Dev Cell.

[9] Yoshii SR, Mizushima N (2017). Monitoring and measuring autophagy. Int J Mol Sci.

[10] Youle RJ, Narendra DP (2011). Mechanisms of mitophagy. Nat Rev Mol Cell Biol.

[11] Hassanpour M, Rezabakhsh A, Rezaie J, Nouri M, Rahbarghazi R (2020). Exosomal cargos modulate autophagy in recipient cells via different signaling pathways. Cell Biosci.

[12] Johansen T, Lamark T (2011). Selective autophagy mediated by autophagic adapter proteins. Autophagy.

[13] Dice JF (1990). Peptide sequences that target cytosolic proteins for lysosomal proteolysis. Trends Biochem Sci.

[14] Tasset I, Cuervo AM (2016). Role of chaperone-mediated autophagy in metabolism. FEBS J.

[15] Bandyopadhyay U, Kaushik S, Varticovski L, Cuervo AM (2008). The chaperone-mediated autophagy receptor organizes in dynamic protein complexes at the lysosomal membrane. Mol Cell Biol.

[16] Kaushik S, Cuervo AM (2018). The coming of age of chaperone-mediated autophagy. Nat Rev Mol Cell Biol.

[17] Schuck S (2020). Microautophagy—distinct molecular mechanisms handle cargoes of many sizes. J Cell Sci.

[18] Oku M, Sakai Y (2018). Three distinct types of microautophagy based on membrane dynamics and molecular machineries. Bioessays.

[19] Sahu R, Kaushik S, Clement CC, Cannizzo ES, Scharf B, Follenzi A, Potolicchio I, Nieves E, Cuervo AM, Santambrogio L (2011). Microautophagy of cytosolic proteins by late endosomes. Dev Cell.

[20] Yarana C, St Clair DK (2017). Chemotherapy-induced tissue injury: an insight into the role of extracellular vesicles-mediated oxidative stress responses. Antioxidants.

[21] Mizushima N, Yoshimori T, Ohsumi Y (2011). The role of Atg proteins in autophagosome formation. Annu Rev Cell Dev Biol.

[22] Lamb CA, Yoshimori T, Tooze SA (2013). The autophagosome: origins unknown, biogenesis complex. Nat Rev Mol Cell Biol.

[23] Suzuki K, Kubota Y, Sekito T, Ohsumi Y (2007). Hierarchy of Atg proteins in pre-autophagosomal structure organization. Genes Cells.

[24] Mizushima N, Noda T, Yoshimori T, Tanaka Y, Ishii T, George MD, Klionsky DJ, Ohsumi M, Ohsumi Y (1998). A protein conjugation system essential for autophagy. Nature.

[25] Ichimura Y, Kirisako T, Takao T, Satomi Y, Shimonishi Y, Ishihara N, Mizushima N, Tanida I, Kominami E, Ohsumi M (2000). A ubiquitin-like system mediates protein lipidation. Nature.

[26] Mizushima N (2010). The role of the Atg1/ULK1 complex in autophagy regulation. Curr Opin Cell Biol.

[27] Ragusa MJ, Stanley RE, Hurley JH (2012). Architecture of the Atg17 complex as a scaffold for autophagosome biogenesis. Cell.

[28] Zachari M, Ganley IG (2017). The mammalian ULK1 complex and autophagy initiation. Essays Biochem.

[29] Alers S, Loffler AS, Wesselborg S, Stork B (2012). Role of AMPK-mTOR-Ulk1/2 in the regulation of autophagy: cross talk, shortcuts, and feedbacks. Mol Cell Biol.

[30] Obara K, Ohsumi Y (2008). Dynamics and function of PtdIns(3)P in autophagy. Autophagy.

[31] Obara K, Ohsumi Y (2011). Atg14: a key player in orchestrating autophagy. Int J Cell Biol.

[32] Liang C, Lee JS, Inn KS, Gack MU, Li Q, Roberts EA, Vergne I, Deretic V, Feng P, Akazawa C (2008). Beclin1-binding UVRAG targets the class C Vps complex to coordinate autophagosome maturation and endocytic trafficking. Nat Cell Biol.

[33] Morris DH, Yip CK, Shi Y, Chait BT, Wang QJ (2015). Beclin 1-Vps34 complex architecture: understanding the nuts and bolts of therapeutic targets. Front Biol.

[34] Kim J, Kim YC, Fang C, Russell RC, Kim JH, Fan W, Liu R, Zhong Q, Guan KL (2013). Differential regulation of distinct Vps34 complexes by AMPK in nutrient stress and autophagy. Cell.

[35] Zhong Y, Wang QJ, Li X, Yan Y, Backer JM, Chait BT, Heintz N, Yue Z (2009). Distinct regulation of autophagic activity by Atg14L and Rubicon associated with Beclin 1-phosphatidylinositol-3-kinase complex. Nat Cell Biol.

[36] Matoba K, Kotani T, Tsutsumi A, Tsuji T, Mori T, Noshiro D, Sugita Y, Nomura N, Iwata S, Ohsumi Y (2020). Atg9 is a lipid scramblase that mediates autophagosomal membrane expansion. Nat Struct Mol Biol.

[37] Sawa-Makarska J, Baumann V, Coudevylle N, von Bulow S, Nogellova V, Abert C, Schuschnig M, Graef M, Hummer G, Martens S (2020). Reconstitution of autophagosome nucleation defines Atg9 vesicles as seeds for membrane formation. Science.

[38] Papinski D, Schuschnig M, Reiter W, Wilhelm L, Barnes CA, Maiolica A, Hansmann I, Pfaffenwimmer T, Kijanska M, Stoffel I (2014). Early steps in autophagy depend on direct phosphorylation of Atg9 by the Atg1 kinase. Mol Cell.

[39] Otomo C, Metlagel Z, Takaesu G, Otomo T (2013). Structure of the human ATG12~ATG5 conjugate required for LC3 lipidation in autophagy. Nat Struct Mol Biol.

[40] Weidberg H, Shvets E, Shpilka T, Shimron F, Shinder V, Elazar Z (2010). LC3 and GATE-16/GABARAP subfamilies are both essential yet act differently in autophagosome biogenesis. EMBO J.

[41] Kabeya Y, Mizushima N, Yamamoto A, Oshitani-Okamoto S, Ohsumi Y, Yoshimori T (2004). LC3, GABARAP and GATE16 localize to autophagosomal membrane depending on form-II formation. J Cell Sci.

[42] Metlagel Z, Otomo C, Takaesu G, Otomo T (2013). Structural basis of ATG3 recognition by the autophagic ubiquitin-like protein ATG12. Proc Natl Acad Sci U S A.

[43] Yu L, Chen Y, Tooze SA (2018). Autophagy pathway: cellular and molecular mechanisms. Autophagy.

[44] Jiang P, Nishimura T, Sakamaki Y, Itakura E, Hatta T, Natsume T, Mizushima N (2014). The HOPS complex mediates autophagosome-lysosome fusion through interaction with syntaxin 17. Mol Biol Cell.

[45] Diao J, Liu R, Rong Y, Zhao M, Zhang J, Lai Y, Zhou Q, Wilz LM, Li J, Vivona S (2015). ATG14 promotes membrane tethering and fusion of autophagosomes to endolysosomes. Nature.

[46] Ung L, Pattamatta U, Carnt N, Wilkinson-Berka JL, Liew G, White AJR (2017). Oxidative stress and reactive oxygen species: a review of their role in ocular disease. Clin Sci (Lond).

[47] Singh A, Kukreti R, Saso L, Kukreti S (2019). Oxidative stress: a key modulator in neurodegenerative diseases. Molecules.

[48] Incalza MA, D'Oria R, Natalicchio A, Perrini S, Laviola L, Giorgino F (2018). Oxidative stress and reactive oxygen species in endothelial dysfunction associated with cardiovascular and metabolic diseases. Vascul Pharmacol.

[49] Shadel GS, Horvath TL (2015). Mitochondrial ROS signaling in organismal homeostasis. Cell.

[50] Murphy MP (2009). How mitochondria produce reactive oxygen species. Biochem J.

[51] Pizzino G, Irrera N, Cucinotta M, Pallio G, Mannino F, Arcoraci V, Squadrito F, Altavilla D, Bitto A (2017). Oxidative stress: harms and benefits for human health. Oxid Med Cell Longev.

[52] Liu PF, Farooqi AA, Peng SY, Yu TJ, Dahms HU, Lee CH, Tang JY, Wang SC, Shu CW, Chang HW (2020). Regulatory effects of noncoding RNAs on the interplay of oxidative stress and autophagy in cancer malignancy and therapy. Semin Cancer Biol.

[53] Filomeni G, Desideri E, Cardaci S, Rotilio G, Ciriolo MR (2010). Under the ROS: thiol network is the principal suspect for autophagy commitment. Autophagy.

[54] Cao J, Schulte J, Knight A, Leslie NR, Zagozdzon A, Bronson R, Manevich Y, Beeson C, Neumann CA (2009). Prdx1 inhibits tumorigenesis via regulating PTEN/AKT activity. EMBO J.

[55] Su Q, Zheng B, Wang CY, Yang YZ, Luo WW, Ma SM, Zhang XH, Ma D, Sun Y, Yang Z (2018). Oxidative stress induces neuronal apoptosis through suppressing transcription factor EB phosphorylation at Ser467. Cell Physiol Biochem.

[56] Kimball SR, Gordon BS, Moyer JE, Dennis MD, Jefferson LS (2016). Leucine induced dephosphorylation of Sestrin2 promotes mTORC1 activation. Cell Signal.

[57] Roberts DJ, Tan-Sah VP, Ding EY, Smith JM, Miyamoto S (2014). Hexokinase-II positively regulates glucose starvation-induced autophagy through TORC1 inhibition. Mol Cell.

[58] Velasco-Miguel S, Buckbinder L, Jean P, Gelbert L, Talbott R, Laidlaw J, Seizinger B, Kley N (1999). PA26, a novel target of the p53 tumor suppressor and member of the GADD family of DNA damage and growth arrest inducible genes. Oncogene.

[59] Shin BY, Jin SH, Cho IJ, Ki SH (2012). Nrf2-ARE pathway regulates induction of Sestrin-2 expression. Free Radic Biol Med.

[60] Shi X, Doycheva DM, Xu L, Tang J, Yan M, Zhang JH (2016). Sestrin2 induced by hypoxia inducible factor1 alpha protects the blood-brain barrier via inhibiting VEGF after severe hypoxic-ischemic injury in neonatal rats. Neurobiol Dis.

[61] Zhang XY, Wu XQ, Deng R, Sun T, Feng GK, Zhu XF (2013). Upregulation of sestrin 2 expression via JNK pathway activation contributes to autophagy induction in cancer cells. Cell Signal.

[62] Saxton RA, Knockenhauer KE, Wolfson RL, Chantranupong L, Pacold ME, Wang T, Schwartz TU, Sabatini DM (2016). Structural basis for leucine sensing by the Sestrin2-mTORC1 pathway. Science.

[63] Kim H, An S, Ro SH, Teixeira F, Park GJ, Kim C, Cho CS, Kim JS, Jakob U, Lee JH (2015). Janus-faced Sestrin2 controls ROS and mTOR signalling through two separate functional domains. Nat Commun.

[64] Cordani M, Sanchez-Alvarez M, Strippoli R, Bazhin AV, Donadelli M (2019). Sestrins at the interface of ROS control and autophagy regulation in health and disease. Oxid Med Cell Longev.

[65] Lim J, Lachenmayer ML, Wu S, Liu W, Kundu M, Wang R, Komatsu M, Oh YJ, Zhao Y, Yue Z (2015). Proteotoxic stress induces phosphorylation of p62/SQSTM1 by ULK1 to regulate selective autophagic clearance of protein aggregates. PLoS Genet.

[66] Lee JH, Budanov AV, Park EJ, Birse R, Kim TE, Perkins GA, Ocorr K, Ellisman MH, Bodmer R, Bier E (2010). Sestrin as a feedback inhibitor of TOR that prevents age-related pathologies. Science.

[67] Scherz-Shouval R, Shvets E, Fass E, Shorer H, Gil L, Elazar Z (2007). Reactive oxygen species are essential for autophagy and specifically regulate the activity of Atg4. EMBO J.

[68] Orenstein SJ, Cuervo AM (2010). Chaperone-mediated autophagy: molecular mechanisms and physiological relevance. Semin Cell Dev Biol.

[69] Zhang L, Wang H, Xu J, Zhu J, Ding K (2014). Inhibition of cathepsin S induces autophagy and apoptosis in human glioblastoma cell lines through ROS-mediated PI3K/AKT/mTOR/p70S6K and JNK signaling pathways. Toxicol Lett.

[70] Zhang J, Kim J, Alexander A, Cai S, Tripathi DN, Dere R, Tee AR, Tait-Mulder J, Di Nardo A, Han JM (2013). A tuberous sclerosis complex signalling node at the peroxisome regulates mTORC1 and autophagy in response to ROS. Nat Cell Biol.

[71] Settembre C, Di Malta C, Polito VA, Garcia Arencibia M, Vetrini F, Erdin S, Erdin SU, Huynh T, Medina D, Colella P (2011). TFEB links autophagy to lysosomal biogenesis. Science.

[72] Wu JJ, Quijano C, Chen E, Liu H, Cao L, Fergusson MM, Rovira II, Gutkind S, Daniels MP, Komatsu M (2009). Mitochondrial dysfunction and oxidative stress mediate the physiological impairment induced by the disruption of autophagy. Aging.

[73] Tal MC, Sasai M, Lee HK, Yordy B, Shadel GS, Iwasaki A (2009). Absence of autophagy results in reactive oxygen species-dependent amplification of RLR signaling. Proc Natl Acad Sci USA.

[74] Filomeni G, De Zio D, Cecconi F (2015). Oxidative stress and autophagy: the clash between damage and metabolic needs. Cell Death Differ.

[75] Gao Q (2019). Oxidative stress and autophagy. Adv Exp Med Biol.

[76] Chen Y, Azad MB, Gibson SB (2009). Superoxide is the major reactive oxygen species regulating autophagy. Cell Death Differ.

[77] Fukai T, Ushio-Fukai M (2011). Superoxide dismutases: role in redox signaling, vascular function, and diseases. Antioxid Redox Signal.

[78] Ribas V, Garcia-Ruiz C, Fernandez-Checa JC (2014). Glutathione and mitochondria. Front Pharmacol.

[79] Forrester SJ, Kikuchi DS, Hernandes MS, Xu Q, Griendling KK (2018). Reactive oxygen species in metabolic and inflammatory signaling. Circ Res.

[80] Yang S, Xia C, Li S, Du L, Zhang L, Zhou R (2014). Defective mitophagy driven by dysregulation of rheb and KIF5B contributes to mitochondrial reactive oxygen species (ROS)-induced nod-like receptor 3 (NLRP3) dependent proinflammatory response and aggravates lipotoxicity. Redox Biol.

[81] Kurihara Y, Kanki T, Aoki Y, Hirota Y, Saigusa T, Uchiumi T, Kang D (2012). Mitophagy plays an essential role in reducing mitochondrial production of reactive oxygen species and mutation of mitochondrial DNA by maintaining mitochondrial quantity and quality in yeast. J Biol Chem.

[82] Wang Y, Nartiss Y, Steipe B, McQuibban GA, Kim PK (2012). ROS-induced mitochondrial depolarization initiates PARK2/PARKIN-dependent mitochondrial degradation by autophagy. Autophagy.

[83] Ge P, Dawson VL, Dawson TM (2020). PINK1 and Parkin mitochondrial quality control: a source of regional vulnerability in Parkinson's disease. Mol Neurodegener.

[84] Sarraf SA, Raman M, Guarani-Pereira V, Sowa ME, Huttlin EL, Gygi SP, Harper JW (2013). Landscape of the PARKIN-dependent ubiquitylome in response to mitochondrial depolarization. Nature.

[85] Rose CM, Isasa M, Ordureau A, Prado MA, Beausoleil SA, Jedrychowski MP, Finley DJ, Harper JW, Gygi SP (2016). Highly multiplexed quantitative mass spectrometry analysis of ubiquitylomes. Cell Syst.

[86] Heo JM, Ordureau A, Paulo JA, Rinehart J, Harper JW (2015). The PINK1-PARKIN mitochondrial ubiquitylation pathway drives a program of OPTN/NDP52 recruitment and TBK1 activation to promote mitophagy. Mol Cell.

[87] Eiyama A, Okamoto K (2015). PINK1/Parkin-mediated mitophagy in mammalian cells. Curr Opin Cell Biol.

[88] Xiao B, Deng X, Lim GGY, Xie S, Zhou ZD, Lim KL, Tan EK (2017). Superoxide drives progression of Parkin/PINK1-dependent mitophagy following translocation of Parkin to mitochondria. Cell Death Dis.

[89] Xiao B, Goh JY, Xiao L, Xian H, Lim KL, Liou YC (2017). Reactive oxygen species trigger Parkin/PINK1 pathway-dependent mitophagy by inducing mitochondrial recruitment of Parkin. J Biol Chem.

[90] Bellot G, Garcia-Medina R, Gounon P, Chiche J, Roux D, Pouyssegur J, Mazure NM (2009). Hypoxia-induced autophagy is mediated through hypoxia-inducible factor induction of BNIP3 and BNIP3L via their BH3 domains. Mol Cell Biol.

[91] Sowter HM, Ratcliffe PJ, Watson P, Greenberg AH, Harris AL (2001). HIF-1-dependent regulation of hypoxic induction of the cell death factors BNIP3 and NIX in human tumors. Cancer Res.

[92] Wanders RJ, Waterham HR (2006). Biochemistry of mammalian peroxisomes revisited. Annu Rev Biochem.

[93] Bonekamp NA, Volkl A, Fahimi HD, Schrader M (2009). Reactive oxygen species and peroxisomes: struggling for balance. BioFactors.

[94] Fransen M, Nordgren M, Wang B, Apanasets O (2012). Role of peroxisomes in ROS/RNS-metabolism: implications for human disease. Biochim Biophys Acta.

[95] Schrader M, Fahimi HD (2006). Peroxisomes and oxidative stress. Biochim Biophys Acta.

[96] Guo Z, Kozlov S, Lavin MF, Person MD, Paull TT (2010). ATM activation by oxidative stress. Science.

[97] Ditch S, Paull TT (2012). The ATM protein kinase and cellular redox signaling: beyond the DNA damage response. Trends Biochem Sci.

[98] Guo Z, Deshpande R, Paull TT (2010). ATM activation in the presence of oxidative stress. Cell Cycle.

[99] Alexander A, Cai SL, Kim J, Nanez A, Sahin M, MacLean KH, Inoki K, Guan KL, Shen J, Person MD (2010). ATM signals to TSC2 in the cytoplasm to regulate mTORC1 in response to ROS. Proc Natl Acad Sci U S A.

[100] Tripathi DN, Chowdhury R, Trudel LJ, Tee AR, Slack RS, Walker CL, Wogan GN (2013). Reactive nitrogen species regulate autophagy through ATM-AMPK-TSC2-mediated suppression of mTORC1. Proc Natl Acad Sci USA.

[101] Zhang J, Tripathi DN, Jing J, Alexander A, Kim J, Powell RT, Dere R, Tait-Mulder J, Lee JH, Paull TT (2015). ATM functions at the peroxisome to induce pexophagy in response to ROS. Nat Cell Biol.

[102] Kamsler A, Daily D, Hochman A, Stern N, Shiloh Y, Rotman G, Barzilai A (2001). Increased oxidative stress in ataxia telangiectasia evidenced by alterations in redox state of brains from Atm-deficient mice. Cancer Res.

[103] Reichenbach J, Schubert R, Schindler D, Muller K, Bohles H, Zielen S (2002). Elevated oxidative stress in patients with ataxia telangiectasia. Antioxid Redox Signal.

[104] Kaushik S, Cuervo AM (2012). Chaperone-mediated autophagy: a unique way to enter the lysosome world. Trends Cell Biol.

[105] Kiffin R, Christian C, Knecht E, Cuervo AM (2004). Activation of chaperone-mediated autophagy during oxidative stress. Mol Biol Cell.

[106] Lee J, Giordano S, Zhang J (2012). Autophagy, mitochondria and oxidative stress: cross-talk and redox signalling. Biochem J.

[107] Callahan MK, Chaillot D, Jacquin C, Clark PR, Menoret A (2002). Differential acquisition of antigenic peptides by Hsp70 and Hsc70 under oxidative conditions. J Biol Chem.

[108] Lee JJ, Ishihara K, Notomi S, Efstathiou NE, Ueta T, Maidana D, Chen X, Iesato Y, Caligiana A, Vavvas DG (2020). Lysosome-associated membrane protein-2 deficiency increases the risk of reactive oxygen species-induced ferroptosis in retinal pigment epithelial cells. Biochem Biophys Res Commun.

[109] Massey AC, Kaushik S, Sovak G, Kiffin R, Cuervo AM (2006). Consequences of the selective blockage of chaperone-mediated autophagy. Proc Natl Acad Sci U S A.

[110] Suzuki T, Yamamoto M (2017). Stress-sensing mechanisms and the physiological roles of the Keap1-Nrf2 system during cellular stress. J Biol Chem.

[111] Suzuki T, Muramatsu A, Saito R, Iso T, Shibata T, Kuwata K, Kawaguchi SI, Iwawaki T, Adachi S, Suda H (2019). Molecular mechanism of cellular oxidative stress sensing by Keap1. Cell Rep.

[112] Friling RS, Bergelson S, Daniel V (1992). Two adjacent AP-1-like binding sites form the electrophile-responsive element of the murine glutathione S-transferase Ya subunit gene. Proc Natl Acad Sci USA.

[113] Rushmore TH, Morton MR, Pickett CB (1991). The antioxidant responsive element. Activation by oxidative stress and identification of the DNA consensus sequence required for functional activity. J Biol Chem.

[114] Telakowski-Hopkins CA, King RG, Pickett CB (1988). Glutathione S-transferase Ya subunit gene: identification of regulatory elements required for basal level and inducible expression. Proc Natl Acad Sci USA.

[115] Pajares M, Jimenez-Moreno N, Garcia-Yague AJ, Escoll M, de Ceballos ML, Van Leuven F, Rabano A, Yamamoto M, Rojo AI, Cuadrado A (2016). Transcription factor NFE2L2/NRF2 is a regulator of macroautophagy genes. Autophagy.

[116] Jain A, Lamark T, Sjottem E, Larsen KB, Awuh JA, Overvatn A, McMahon M, Hayes JD, Johansen T (2010). p62/SQSTM1 is a target gene for transcription factor NRF2 and creates a positive feedback loop by inducing antioxidant response element-driven gene transcription. J Biol Chem.

[117] Kaspar JW, Niture SK, Jaiswal AK (2009). Nrf 2:INrf2 (Keap1) signaling in oxidative stress. Free Radic Biol Med.

[118] Ichimura Y, Waguri S, Sou YS, Kageyama S, Hasegawa J, Ishimura R, Saito T, Yang Y, Kouno T, Fukutomi T (2013). Phosphorylation of p62 activates the Keap1-Nrf2 pathway during selective autophagy. Mol Cell.

[119] Kageyama S, Gudmundsson SR, Sou YS, Ichimura Y, Tamura N, Kazuno S, Ueno T, Miura Y, Noshiro D, Abe M (2021). p62/SQSTM1-droplet serves as a platform for autophagosome formation and anti-oxidative stress response. Nat Commun.

[120] Kumar RR, Narasimhan M, Shanmugam G, Hong J, Devarajan A, Palaniappan S, Zhang J, Halade GV, Darley-Usmar VM, Hoidal JR (2016). Abrogation of Nrf2 impairs antioxidant signaling and promotes atrial hypertrophy in response to high-intensity exercise stress. J Transl Med.

[121] Georgakopoulos ND, Frison M, Alvarez MS, Bertrand H, Wells G, Campanella M (2017). Reversible Keap1 inhibitors are preferential pharmacological tools to modulate cellular mitophagy. Sci Rep.

[122] Murata H, Takamatsu H, Liu S, Kataoka K, Huh NH, Sakaguchi M (2015). NRF2 regulates PINK1 expression under oxidative stress conditions. PLoS ONE.

[123] Yamada T, Murata D, Adachi Y, Itoh K, Kameoka S, Igarashi A, Kato T, Araki Y, Huganir RL, Dawson TM (2018). Mitochondrial stasis reveals p62-mediated ubiquitination in parkin-independent mitophagy and mitigates nonalcoholic fatty liver disease. Cell Metab.

[124] Bialik S, Dasari SK, Kimchi A (2018). Autophagy-dependent cell death—where, how and why a cell eats itself to death. J Cell Sci.

[125] Chen Y, McMillan-Ward E, Kong J, Israels SJ, Gibson SB (2008). Oxidative stress induces autophagic cell death independent of apoptosis in transformed and cancer cells. Cell Death Differ.

[126] Chen Y, McMillan-Ward E, Kong J, Israels SJ, Gibson SB (2007). Mitochondrial electron-transport-chain inhibitors of complexes I and II induce autophagic cell death mediated by reactive oxygen species. J Cell Sci.

[127] Ha S, Ryu HY, Chung KM, Baek SH, Kim EK, Yu SW (2015). Regulation of autophagic cell death by glycogen synthase kinase-3beta in adult hippocampal neural stem cells following insulin withdrawal. Mol Brain.

[128] Law BYK, Michelangeli F, Qu YQ, Xu SW, Han Y, Mok SWF, Dias I, Javed MU, Chan WK, Xue WW (2019). Neferine induces autophagy-dependent cell death in apoptosis-resistant cancers via ryanodine receptor and Ca(2+)-dependent mechanism. Sci Rep.

[129] Quigley HA, Broman AT (2006). The number of people with glaucoma worldwide in 2010 and 2020. Br J Ophthalmol.

[130] Nettesheim A, Dixon A, Shim MS, Coyne A, Walsh M, Liton PB (2020). Autophagy in the aging and experimental ocular hypertensive mouse model. Invest Ophthalmol Vis Sci.

[131] Lin WJ, Kuang HY (2014). Oxidative stress induces autophagy in response to multiple noxious stimuli in retinal ganglion cells. Autophagy.

[132] Amankwa CE, Gondi SR, Dibas A, Weston C, Funk A, Nguyen T, Nguyen KT, Ellis DZ, Acharya S (2021). Novel thiol containing hybrid antioxidant-nitric oxide donor small molecules for treatment of glaucoma. Antioxidants.

[133] Izzotti A, Bagnis A, Sacca SC (2006). The role of oxidative stress in glaucoma. Mutat Res.

[134] Shim MS, Nettesheim A, Dixon A, Liton PB (2021). Primary cilia and the reciprocal activation of AKT and SMAD2/3 regulate stretch-induced autophagy in trabecular meshwork cells. Proc Natl Acad Sci USA.

[135] Zuo L, Khan RS, Lee V, Dine K, Wu W, Shindler KS (2013). SIRT1 promotes RGC survival and delays loss of function following optic nerve crush. Invest Ophthalmol Vis Sci.

[136] Kang LH, Zhang S, Jiang S, Hu N (2019). Activation of autophagy in the retina after optic nerve crush injury in rats. Int J Ophthalmol.

[137] Zhan Z, Wu Y, Liu Z, Quan Y, Li D, Huang Y, Yang S, Wu K, Huang L, Yu M (2020). Reduced dendritic spines in the visual cortex contralateral to the optic nerve crush eye in adult mice. Invest Ophthalmol Vis Sci.

[138] Kim SH, Munemasa Y, Kwong JM, Ahn JH, Mareninov S, Gordon LK, Caprioli J, Piri N (2008). Activation of autophagy in retinal ganglion cells. J Neurosci Res.

[139] Knoferle J, Koch JC, Ostendorf T, Michel U, Planchamp V, Vutova P, Tonges L, Stadelmann C, Bruck W, Bahr M (2010). Mechanisms of acute axonal degeneration in the optic nerve in vivo. Proc Natl Acad Sci USA.

[140] Rodriguez-Muela N, Germain F, Marino G, Fitze PS, Boya P (2012). Autophagy promotes survival of retinal ganglion cells after optic nerve axotomy in mice. Cell Death Differ.

[141] Porter K, Hirt J, Stamer WD, Liton PB (2015). Autophagic dysregulation in glaucomatous trabecular meshwork cells. Biochim Biophys Acta.

[142] Kitaoka Y, Sase K, Tsukahara C, Fujita N, Tokuda N, Kogo J, Takagi H (2020). Axonal protection by a small molecule SIRT1 activator, SRT2104, with alteration of autophagy in TNF-induced optic nerve degeneration. Jpn J Ophthalmol.

[143] Yazdankhah M, Ghosh S, Shang P, Stepicheva N, Hose S, Liu H, Chamling X, Tian S, Sullivan MLG, Calderon MJ (2021). BNIP3L-mediated mitophagy is required for mitochondrial remodeling during the differentiation of optic nerve oligodendrocytes. Autophagy.

[144] Beckers A, Vanhunsel S, Van Dyck A, Bergmans S, Masin L, Moons L (2021). Injury-induced autophagy delays axonal regeneration after optic nerve damage in adult zebrafish. Neuroscience.

[145] Rodriguez-Muela N, Boya P (2012). Axonal damage, autophagy and neuronal survival. Autophagy.

[146] Ying H, Yue BY (2016). Optineurin: the autophagy connection. Exp Eye Res.

[147] Zhang S, Shao Z, Liu X, Hou M, Cheng F, Lei D, Yuan H (2021). The E50K optineurin mutation impacts autophagy-mediated degradation of TDP-43 and leads to RGC apoptosis in vivo and in vitro. Cell Death Discov.

[148] Losiewicz MK, Elghazi L, Fingar DC, Rajala RVS, Lentz SI, Fort PE, Abcouwer SF, Gardner TW (2020). mTORC1 and mTORC2 expression in inner retinal neurons and glial cells. Exp Eye Res.

[149] Russo R, Berliocchi L, Adornetto A, Amantea D, Nucci C, Tassorelli C, Morrone LA, Bagetta G, Corasaniti MT (2013). In search of new targets for retinal neuroprotection: is there a role for autophagy?. Curr Opin Pharmacol.

[150] Russo R, Varano GP, Adornetto A, Nazio F, Tettamanti G, Girardello R, Cianfanelli V, Cavaliere F, Morrone LA, Corasaniti MT (2018). Rapamycin and fasting sustain autophagy response activated by ischemia/reperfusion injury and promote retinal ganglion cell survival. Cell Death Dis.

[151] Bell K, Rosignol I, Sierra-Filardi E, Rodriguez-Muela N, Schmelter C, Cecconi F, Grus F, Boya P (2020). Age related retinal Ganglion cell susceptibility in context of autophagy deficiency. Cell Death Discov.

[152] Kauppinen A (2020). Introduction to the multi-author review on macular degeneration. Cell Mol Life Sci.

[153] Fleckenstein M, Keenan TDL, Guymer RH, Chakravarthy U, Schmitz-Valckenberg S, Klaver CC, Wong WT, Chew EY (2021). Age-related macular degeneration. Nat Rev Dis Primers.

[154] Chan CM, Huang DY, Sekar P, Hsu SH, Lin WW (2019). Reactive oxygen species-dependent mitochondrial dynamics and autophagy confer protective effects in retinal pigment epithelial cells against sodium iodate-induced cell death. J Biomed Sci.

[155] Zhang ZY, Bao XL, Cong YY, Fan B, Li GY (2020). Autophagy in age-related macular degeneration: a regulatory mechanism of oxidative stress. Oxid Med Cell Longev.

[156] Wang S, Ji LY, Li L, Li JM (2019). Oxidative stress, autophagy and pyroptosis in the neovascularization of oxygeninduced retinopathy in mice. Mol Med Rep.

[157] Song C, Mitter SK, Qi X, Beli E, Rao HV, Ding J, Ip CS, Gu H, Akin D, Dunn WA (2017). Oxidative stress-mediated NFkappaB phosphorylation upregulates p62/SQSTM1 and promotes retinal pigmented epithelial cell survival through increased autophagy. PLoS ONE.

[158] Blasiak J, Szczepanska J, Fila M, Pawlowska E, Kaarniranta K (2021). Potential of telomerase in age-related macular degeneration-involvement of Senescence, DNA damage response and autophagy and a key role of PGC-1alpha. Int J Mol Sci.

[159] Yang X, Pan X, Zhao X, Luo J, Xu M, Bai D, Hu Y, Liu X, Yu Q, Gao D (2019). Autophagy and age-related eye diseases. Biomed Res Int.

[160] George SM, Lu F, Rao M, Leach LL, Gross JM (2021). The retinal pigment epithelium: Development, injury responses, and regenerative potential in mammalian and non-mammalian systems. Prog Retin Eye Res.

[161] Kaarniranta K, Tokarz P, Koskela A, Paterno J, Blasiak J (2017). Autophagy regulates death of retinal pigment epithelium cells in age-related macular degeneration. Cell Biol Toxicol.

[162] Liu J, Copland DA, Theodoropoulou S, Chiu HA, Barba MD, Mak KW, Mack M, Nicholson LB, Dick AD (2016). Impairing autophagy in retinal pigment epithelium leads to inflammasome activation and enhanced macrophage-mediated angiogenesis. Sci Rep.

[163] Wang AL, Lukas TJ, Yuan M, Du N, Tso MO, Neufeld AH (2009). Autophagy and exosomes in the aged retinal pigment epithelium: possible relevance to drusen formation and age-related macular degeneration. PLoS ONE.

[164] Rodriguez-Muela N, Koga H, Garcia-Ledo L, de la Villa P, de la Rosa EJ, Cuervo AM, Boya P (2013). Balance between autophagic pathways preserves retinal homeostasis. Aging Cell.

[165] Mitter SK, Song C, Qi X, Mao H, Rao H, Akin D, Lewin A, Grant M, Dunn W, Ding J (2014). Dysregulated autophagy in the RPE is associated with increased susceptibility to oxidative stress and AMD. Autophagy.

[166] Golestaneh N, Chu Y, Xiao YY, Stoleru GL, Theos AC (2017). Dysfunctional autophagy in RPE, a contributing factor in age-related macular degeneration. Cell Death Dis.

[167] Szatmari-Toth M, Kristof E, Vereb Z, Akhtar S, Facsko A, Fesus L, Kauppinen A, Kaarniranta K, Petrovski G (2016). Clearance of autophagy-associated dying retinal pigment epithelial cells—a possible source for inflammation in age-related macular degeneration. Cell Death Dis.

[168] Szatmari-Toth M, Ilmarinen T, Mikhailova A, Skottman H, Kauppinen A, Kaarniranta K, Kristof E, Lytvynchuk L, Vereb Z, Fesus L (2019). Human embryonic stem cell-derived retinal pigment epithelium-role in dead cell clearance and inflammation. Int J Mol Sci.

[169] Sheu SJ, Chen JL, Bee YS, Lin SH, Shu CW (2019). ERBB2-modulated ATG4B and autophagic cell death in human ARPE19 during oxidative stress. PLoS ONE.

[170] Chang KC, Snow A, LaBarbera DV, Petrash JM (2015). Aldose reductase inhibition alleviates hyperglycemic effects on human retinal pigment epithelial cells. Chem Biol Interact.

[171] Yao J, Tao ZF, Li CP, Li XM, Cao GF, Jiang Q, Yan B (2014). Regulation of autophagy by high glucose in human retinal pigment epithelium. Cell Physiol Biochem.

[172] Piano I, Novelli E, Della Santina L, Strettoi E, Cervetto L, Gargini C (2016). Involvement of autophagic pathway in the progression of retinal degeneration in a mouse model of diabetes. Front Cell Neurosci.

[173] Coucha M, Elshaer SL, Eldahshan WS, Mysona BA, El-Remessy AB (2015). Molecular mechanisms of diabetic retinopathy: potential therapeutic targets. Middle East Afr J Ophthalmol.

[174] Volpe CMO, Villar-Delfino PH, Dos Anjos PMF, Nogueira-Machado JA (2018). Cellular death, reactive oxygen species (ROS) and diabetic complications. Cell Death Dis.

[175] Zhou R, Yazdi AS, Menu P, Tschopp J (2011). A role for mitochondria in NLRP3 inflammasome activation. Nature.

[176] Rosa MD, Distefano G, Gagliano C, Rusciano D, Malaguarnera L (2016). Autophagy in diabetic retinopathy. Curr Neuropharmacol.

[177] Fu D, Wu M, Zhang J, Du M, Yang S, Hammad SM, Wilson K, Chen J, Lyons TJ (2012). Mechanisms of modified LDL-induced pericyte loss and retinal injury in diabetic retinopathy. Diabetologia.

[178] Aihara M (2021). Prostanoid receptor agonists for glaucoma treatment. Jpn J Ophthalmol.

[179] Skov AG, Rives AS, Freiberg J, Virgili G, Azuara-Blanco A, Kolko M: Comparative efficacy and safety of preserved versus preservative-free beta-blockers in patients with glaucoma or ocular hypertension: a systematic review. Acta Ophthalmol 2021. 10.1111/aos.14926 34128326

[180] Nocentini A, Supuran CT (2019). Adrenergic agonists and antagonists as antiglaucoma agents: a literature and patent review (2013–2019). Expert Opin Ther Pat.

[181] Jansook P, Hnin HM, Loftsson T, Stefansson E (2021). Cyclodextrin-based formulation of carbonic anhydrase inhibitors for ocular delivery—a review. Int J Pharm.

[182] Al-Humimat G, Marashdeh I, Daradkeh D, Kooner K (2021). Investigational Rho kinase inhibitors for the treatment of glaucoma. J Exp Pharmacol.

[183] Faiq MA, Wollstein G, Schuman JS, Chan KC (2019). Cholinergic nervous system and glaucoma: from basic science to clinical applications. Prog Retin Eye Res.

[184] Toteberg-Harms M, Meier-Gibbons F (2021). Is laser trabeculoplasty the new star in glaucoma treatment?. Curr Opin Ophthalmol.

[185] Wolters JEJ, van Mechelen RJS, Al Majidi R, Pinchuk L, Webers CAB, Beckers HJM, Gorgels T (2021). History, presence, and future of mitomycin C in glaucoma filtration surgery. Curr Opin Ophthalmol.

[186] Riva I, Roberti G, Katsanos A, Oddone F, Quaranta L (2017). A Review of the ahmed glaucoma valve implant and comparison with other surgical operations. Adv Ther.

[187] Kan JT, Betzler BK, Lim SY, Ang BCH (2021). Anterior segment imaging in minimally invasive glaucoma surgery—a systematic review. Acta Ophthalmol.

[188] Chang KC, Sun C, Cameron EG, Madaan A, Wu S, Xia X, Zhang X, Tenerelli K, Nahmou M, Knasel CM (2019). Opposing effects of growth and differentiation factors in cell-fate specification. Curr Biol.

[189] Zhang X, Tenerelli K, Wu S, Xia X, Yokota S, Sun C, Galvao J, Venugopalan P, Li C, Madaan A (2020). Cell transplantation of retinal ganglion cells derived from hESCs. Restor Neurol Neurosci.

[190] Fligor CM, Langer KB, Sridhar A, Ren Y, Shields PK, Edler MC, Ohlemacher SK, Sluch VM, Zack DJ, Zhang C (2018). Three-dimensional retinal organoids facilitate the investigation of retinal ganglion cell development, organization and neurite outgrowth from human pluripotent stem cells. Sci Rep.

[191] Sluch VM, Chamling X, Liu MM, Berlinicke CA, Cheng J, Mitchell KL, Welsbie DS, Zack DJ (2017). Enhanced stem cell differentiation and immunopurification of genome engineered human retinal ganglion cells. Stem Cells Transl Med.

[192] Luo Z, Xian B, Li K, Li K, Yang R, Chen M, Xu C, Tang M, Rong H, Hu D (2021). scaffolds facilitate epiretinal transplantation of hiPSC-derived retinal neurons in nonhuman primates. Acta Biomater.

[193] Miltner AM, La Torre A (2019). Retinal ganglion cell replacement: current status and challenges ahead. Dev Dyn.

[194] Moore DL, Blackmore MG, Hu Y, Kaestner KH, Bixby JL, Lemmon VP, Goldberg JL (2009). KLF family members regulate intrinsic axon regeneration ability. Science.

[195] Park KK, Liu K, Hu Y, Smith PD, Wang C, Cai B, Xu B, Connolly L, Kramvis I, Sahin M (2008). Promoting axon regeneration in the adult CNS by modulation of the PTEN/mTOR pathway. Science.

[196] Chang KC, Bian M, Xia X, Madaan A, Sun C, Wang Q, Li L, Nahmou M, Noro T, Yokota S (2021). Posttranslational modification of Sox11 regulates RGC survival and axon regeneration. eNeuro.

[197] Xie L, Yin Y, Benowitz L (2021). Chemokine CCL5 promotes robust optic nerve regeneration and mediates many of the effects of CNTF gene therapy. Proc Natl Acad Sci USA.

[198] Patel AK, Broyer RM, Lee CD, Lu T, Louie MJ, La Torre A, Al-Ali H, Vu MT, Mitchell KL, Wahlin KJ (2020). Inhibition of GCK-IV kinases dissociates cell death and axon regeneration in CNS neurons. Proc Natl Acad Sci USA.

[199] Williams PR, Benowitz LI, Goldberg JL, He Z (2020). Axon regeneration in the mammalian optic nerve. Annu Rev Vis Sci.

[200] Matloob S, Fan JC, Danesh-Meyer HV (2011). Multifocal malignant optic glioma of adulthood presenting as acute anterior optic neuropathy. J Clin Neurosci.

[201] Tooley AA, Rasool N, Campbell A, Kazim M (2021). Acute angle plication of optic nerve glioma as a mechanism of rapidly progressive visual loss. Orbit.

[202] Pan Y, Hysinger JD, Barron T, Schindler NF, Cobb O, Guo X, Yalcin B, Anastasaki C, Mulinyawe SB, Ponnuswami A (2021). NF1 mutation drives neuronal activity-dependent initiation of optic glioma. Nature.

[203] Brown EE, DeWeerd AJ, Ildefonso CJ, Lewin AS, Ash JD (2019). Mitochondrial oxidative stress in the retinal pigment epithelium (RPE) led to metabolic dysfunction in both the RPE and retinal photoreceptors. Redox Biol.

[204] Skrzypczak T, Jany A, Bugajska-Abramek E, Boguslawska J, Kowal-Lange A (2021). A comparative study of ranibizumab and aflibercept for neovascular age-related macular degeneration: 12-month outcomes of Polish therapeutic program in non-tertiary institution. Cureus.

[205] Banaee T, Alwan S, Kellogg C, Kornblau I, El-Annan J (2021). PRN treatment of neovascular AMD with cycles of three monthly injections. J Ophthalmic Vis Res.

[206] Parravano M, Costanzo E, Scondotto G, Trifiro G, Virgili G (2021). Anti-VEGF and other novel therapies for neovascular age-related macular degeneration: an update. BioDrugs.

[207] Chichan H, Maus M, Heindl LM (2021). Subthreshold nanosecond laser, from trials to real-life clinical practice: a cohort study. Clin Ophthalmol.

[208] Gao Y, Yu T, Zhang Y, Dang G (2018). Anti-VEGF monotherapy versus photodynamic therapy and anti-VEGF combination treatment for neovascular age-related macular degeneration: a meta-analysis. Invest Ophthalmol Vis Sci.

[209] Bikbov MM, Orenburkina OI, Babushkin AE, Burkhanov YK (2020). Use of macular lenses in patients with age-related macular degeneration. Vestn Oftalmol.

[210] Arrigo A, Bandello F (2021). Molecular features of classic retinal drugs, retinal therapeutic targets and emerging treatments. Pharmaceutics.

[211] Berrocal MH, Acaba-Berrocal L (2021). Early pars plana vitrectomy for proliferative diabetic retinopathy: update and review of current literature. Curr Opin Ophthalmol.

[212] Chang KC, Petrash JM (2018). Aldo-Keto reductases: multifunctional proteins as therapeutic targets in diabetes and inflammatory disease. Adv Exp Med Biol.

[213] Sonowal H, Ramana KV (2021). Development of aldose reductase inhibitors for the treatment of inflammatory disorders and cancer: current drug design strategies and future directions. Curr Med Chem.

[214] Suzen S, Buyukbingol E (2003). Recent studies of aldose reductase enzyme inhibition for diabetic complications. Curr Med Chem.

[215] Gabbay KH (2004). Aldose reductase inhibition in the treatment of diabetic neuropathy: where are we in 2004?. Curr Diab Rep.

[216] Ishikawa M, Takaseki S, Yoshitomi T, Covey DF, Zorumski CF, Izumi Y (2021). The neurosteroid allopregnanolone protects retinal neurons by effects on autophagy and GABRs/GABAA receptors in rat glaucoma models. Autophagy.

[217] Sbardella D, Tundo GR, Coletta M, Manni G, Oddone F (2021). Dexamethasone downregulates autophagy through accelerated turn-over of the Ulk-1 complex in a trabecular meshwork cells strain: insights on steroid-induced glaucoma pathogenesis. Int J Mol Sci.

[218] Hamano T, Shirafuji N, Yen SH, Yoshida H, Kanaan NM, Hayashi K, Ikawa M, Yamamura O, Fujita Y, Kuriyama M (2020). Rho-kinase ROCK inhibitors reduce oligomeric tau protein. Neurobiol Aging.

[219] Kitaoka Y, Sase K, Tsukahara C, Kojima K, Shiono A, Kogo J, Tokuda N, Takagi H (2017). Axonal protection by ripasudil, a rho kinase inhibitor, via modulating autophagy in TNF-induced optic nerve degeneration. Invest Ophthalmol Vis Sci.

[220] Zhang J, Bai Y, Huang L, Qi Y, Zhang Q, Li S, Wu Y, Li X (2015). Protective effect of autophagy on human retinal pigment epithelial cells against lipofuscin fluorophore A2E: implications for age-related macular degeneration. Cell Death Dis.

[221] Ran Z, Zhang Y, Wen X, Ma J (2019). Curcumin inhibits high glucoseinduced inflammatory injury in human retinal pigment epithelial cells through the ROSPI3K/AKT/mTOR signaling pathway. Mol Med Rep.

[222] Su W, Li Z, Jia Y, Zhuo Y (2014). Rapamycin is neuroprotective in a rat chronic hypertensive glaucoma model. PLoS ONE.

[223] Dai Y, Zheng K, Clark J, Swerdlow RH, Pulst SM, Sutton JP, Shinobu LA, Simon DK (2014). Rapamycin drives selection against a pathogenic heteroplasmic mitochondrial DNA mutation. Hum Mol Genet.

[224] Cinik R, Yuksel N, Pirhan D, Aslan MS, Subasi C, Karaoz E (2016). The effect of everolimus on scar formation in glaucoma filtering surgery in a rabbit model. Curr Eye Res.

[225] Matsuki M, Adachi Y, Ozawa Y, Kimura T, Hoshi T, Okamoto K, Tohyama O, Mitsuhashi K, Yamaguchi A, Matsui J (2017). Targeting of tumor growth and angiogenesis underlies the enhanced antitumor activity of lenvatinib in combination with everolimus. Cancer Sci.

[226] Ambati J, Fowler BJ (2012). Mechanisms of age-related macular degeneration. Neuron.

[227] Duh EJ, Sun JK, Stitt AW (2017). Diabetic retinopathy: current understanding, mechanisms, and treatment strategies. JCI Insight.

[228] Uchida J, Iwai T, Nakatani T (2018). Introduction of everolimus in kidney transplant recipients at a late posttransplant stage. World J Transpl.

[229] Touhami S, Arzouk N, Darugar A, Heron E, Clarencon F, Bodaghi B, LeHoang P, Barrou B, Touitou V (2014). Everolimus-induced posterior reversible encephalopathy syndrome and bilateral optic neuropathy after kidney transplantation. Transplantation.

[230] Canovai E, Cassiman C, Ceulemans LJ, Demaerel P, Sainz-Barriga M, Jochmans I, Monbaliu D, Pirenne J, Vanuytsel T (2020). Tacrolimus-induced optic neuropathy after multivisceral transplantation. Transpl Direct.

[231] Liegl R, Koenig S, Siedlecki J, Haritoglou C, Kampik A, Kernt M (2014). Temsirolimus inhibits proliferation and migration in retinal pigment epithelial and endothelial cells via mTOR inhibition and decreases VEGF and PDGF expression. PLoS ONE.

[232] Jacot JL, Sherris D (2011). Potential therapeutic roles for inhibition of the PI3K/Akt/mTOR pathway in the pathophysiology of diabetic retinopathy. J Ophthalmol.

[233] Kourelis TV, Siegel RD (2012). Metformin and cancer: new applications for an old drug. Med Oncol.

[234] Brown EE, Ball JD, Chen Z, Khurshid GS, Prosperi M, Ash JD (2019). The common antidiabetic drug metformin reduces odds of developing age-related macular degeneration. Invest Ophthalmol Vis Sci.

[235] Lin HC, Stein JD, Nan B, Childers D, Newman-Casey PA, Thompson DA, Richards JE (2015). Association of geroprotective effects of metformin and risk of open-angle glaucoma in persons with diabetes mellitus. JAMA Ophthalmol.

[236] Kim YS, Kim M, Choi MY, Lee DH, Roh GS, Kim HJ, Kang SS, Cho GJ, Kim SJ, Yoo JM (2017). Metformin protects against retinal cell death in diabetic mice. Biochem Biophys Res Commun.

[237] Motoi Y, Shimada K, Ishiguro K, Hattori N (2014). Lithium and autophagy. ACS Chem Neurosci.

[238] Sun XB, Lu HE, Chen Y, Fan XH, Tong B (2014). Effect of lithium chloride on endoplasmic reticulum stress-related PERK/ROCK signaling in a rat model of glaucoma. Pharmazie.

[239] Zeilbeck LF, Muller B, Knobloch V, Tamm ER, Ohlmann A (2014). Differential angiogenic properties of lithium chloride in vitro and in vivo. PLoS ONE.

[240] Ruiz-Pesini E, Emperador S, Lopez-Gallardo E, Hernandez-Ainsa C, Montoya J (2018). Increasing mtDNA levels as therapy for mitochondrial optic neuropathies. Drug Discov Today.

[241] Fauzi YR, Nakahata S, Chilmi S, Ichikawa T, Nueangphuet P, Yamaguchi R, Nakamura T, Shimoda K, Morishita K (2021). Antitumor effects of chloroquine/hydroxychloroquine mediated by inhibition of the NF-kappaB signaling pathway through abrogation of autophagic p47 degradation in adult T-cell leukemia/lymphoma cells. PLoS ONE.

[242] Zhang ML, Zhao GL, Hou Y, Zhong SM, Xu LJ, Li F, Niu WR, Yuan F, Yang XL, Wang Z (2020). Rac1 conditional deletion attenuates retinal ganglion cell apoptosis by accelerating autophagic flux in a mouse model of chronic ocular hypertension. Cell Death Dis.

[243] Kan E, Yakar K, Demirag MD, Gok M (2018). Macular ganglion cell-inner plexiform layer thickness for detection of early retinal toxicity of hydroxychloroquine. Int Ophthalmol.

[244] Reichel C, Berlin A, Radun V, Tarau IS, Hillenkamp J, Kleefeldt N, Sloan KR, Ach T (2020). Quantitative fundus autofluorescence in systemic chloroquine/hydroxychloroquine therapy. Transl Vis Sci Technol.

[245] Singh DK, Muhieddine L, Einstadter D, Ballou S (2019). Incidence of blindness in a population of rheumatic patients treated with hydroxychloroquine. Rheumatol Adv Pract.

[246] Amato R, Catalani E, Dal Monte M, Cammalleri M, Di Renzo I, Perrotta C, Cervia D, Casini G (2018). Autophagy-mediated neuroprotection induced by octreotide in an ex vivo model of early diabetic retinopathy. Pharmacol Res.

[247] Dasgupta B, Yi Y, Chen DY, Weber JD, Gutmann DH (2005). Proteomic analysis reveals hyperactivation of the mammalian target of rapamycin pathway in neurofibromatosis 1-associated human and mouse brain tumors. Cancer Res.

[248] Johannessen CM, Reczek EE, James MF, Brems H, Legius E, Cichowski K (2005). The NF1 tumor suppressor critically regulates TSC2 and mTOR. Proc Natl Acad Sci USA.

[249] Fujita Y, Inagaki N (2017). Metformin: clinical topics and new mechanisms of action. Diabetol Int.

[250] Rena G, Hardie DG, Pearson ER (2017). The mechanisms of action of metformin. Diabetologia.

[251] Meng D, Frank AR, Jewell JL (2018). mTOR signaling in stem and progenitor cells. Development.

